# Surface Smoothing by Atomic Layer Deposition and Etching for the Fabrication of Nanodevices

**DOI:** 10.1021/acsanm.2c04025

**Published:** 2022-11-28

**Authors:** Sven H. Gerritsen, Nicholas J. Chittock, Vincent Vandalon, Marcel A. Verheijen, Harm C. M. Knoops, Wilhelmus M. M. Kessels, Adriaan J. M. Mackus

**Affiliations:** † Department of Applied Physics, Eindhoven University of Technology, P.O. Box 513, 5600MBEindhoven, The Netherlands; ‡ Eurofins Materials Science, High Tech Campus 11, 5656AEEindhoven, The Netherlands; § Oxford Instruments Plasma Technology, North End, BristolBS49 4AP, U.K.

**Keywords:** atomic layer deposition, atomic layer etching, ultrathin films, surface smoothing, plasma processing

## Abstract

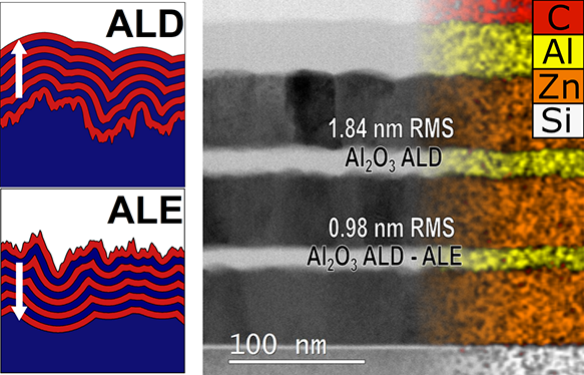

In many nano(opto)electronic devices, the roughness at surfaces and interfaces is of increasing importance, with roughness often contributing toward losses and defects, which can lead to device failure. Consequently, approaches that either limit roughness or smoothen surfaces are required to minimize surface roughness during fabrication. The atomic-scale processing techniques atomic layer deposition (ALD) and atomic layer etching (ALE) have experimentally been shown to smoothen surfaces, with the added benefit of offering uniform and conformal processing and precise thickness control. However, the mechanisms which drive smoothing during ALD and ALE have not been investigated in detail. In this work, smoothing of surfaces by ALD and ALE is studied using finite difference simulations that describe deposition/etching as a front propagating uniformly and perpendicular to the surface at every point. This uniform front propagation model was validated by performing ALD of amorphous Al_2_O_3_ using the TMA/O_2_ plasma. ALE from the TMA/SF_6_ plasma was also studied and resulted in faster smoothing than predicted by purely considering uniform front propagation. Correspondingly, it was found that for such an ALE process, a second mechanism contributes to the smoothing, hypothesized to be related to curvature-dependent surface fluorination. Individually, the atomic-scale processing techniques enable smoothing; however, ALD and ALE will need to be combined to achieve thin and smooth films, as is demonstrated and discussed in this work for multiple applications.

## 1 Introduction

With the semiconductor industry pursuing ever improved device performance and yield, the quality of surfaces and interfaces plays an increasingly large role, with nanoscale surface roughness often being a limiting parameter in final device performance. Roughness at interfaces can act as a nucleation point for defects, introduce nonuniformities, and contribute to trap states.^1−10^ For example, in both FinFETs and gate-all-around FETs, roughness leads to variability of the threshold voltage due to deviating gate lengths, while roughness in the fins/nanowires reduces the carrier mobility.^1,5−7^ Metal–insulator–metal (MIM) capacitor structures in radiofrequency and analog applications are detrimentally affected by roughness, causing nonuniform electric fields^10^ and consequently an increased leakage current and electronic noise.^3,8,9^ In the field of photonics, sidewall roughness in optical waveguides has been shown to cause scattering losses.^11,12^ This is just a small selection of applications where controlling surface roughness is vital to improve final device performance.

The manufacturing of integrated circuits requires several patterning, deposition, and etching steps, all of which can contribute to the development of roughness. Most of the reported sources of roughness introduction are associated with film crystallization,^13^ the random nature of processes,^14^ or variability in the reactant exposure.^15,16^ Previous approaches to mitigate surface roughness have often aimed at preventing roughness from forming in the first place. An alternative approach is to actively reduce roughness using a dedicated process step. As certain films have an intrinsic roughness that cannot be reduced further during their deposition, a post-deposition smoothing step is in some cases the only option.^16,17^

In the fabrication of current and future nanoelectronics, atomic layer deposition (ALD) and atomic layer etching (ALE) are enabling steps because of their atomic-level thickness control, combined by their inherent uniformity and conformality. Both ALD and ALE are based on two (or more) sequential self-limiting half-reactions, resulting in the final film thickness being dependent on the number of cycles performed. Increasingly, these atomic-scale processes are being utilized to deposit a wide variety of films for a growing number of applications.^18−23^

Smoothing by ALD has been observed in several cases, especially when depositing an amorphous material on top of a rough (e.g., polycrystalline) material.^17,24−28^ However, such smoothing effects have only been reported for relatively thick layers. For example, Elam et al. measured a reduction in root-mean-square (RMS) roughness of 5 × 10^–5^ nm/cycle, requiring over 1000 cycles of ALD Al_2_O_3_ for significant smoothing of a ZnO surface.^17^ Similarly, Myers et al. found a reduction of 6 × 10^–4^ nm/cycle for ALD Al_2_O_3_ on rough Si substrates.^28^ The observed smoothing has been attributed to the conformal nature of ALD. Based on geometrical considerations and finite difference simulations, conformal deposition does not necessarily mean that the surfaces are perfectly reproduced.^27,29−31^ Instead, valley walls grow together and fill small features, leading to them being smoothed out.^27^

Interestingly, more significant smoothing effects have been observed in recent ALE studies for both isotropic and anisotropic processes.^32−34^ For example, Zywotko et al. reported a reduction of RMS surface roughness of 0.63 to 0.37 nm during 100 cycles of isotropic Al_2_O_3_ ALE with hydrogen fluoride and TMA, corresponding to 3 × 10^–3^ nm/cycle smoothing on average.^34^ Smoothing by isotropic ALE is hypothesized to be caused by the isotropic nature of the process, similar to ALD smoothing. However, the faster smoothing rate as compared to ALD requires further investigation and suggests that there is an additional mechanism contributing to smoothing.

The isotropic ALE process studied in this work involves fluorination and ligand-exchange reactions: a fluorine-containing plasma modifies a surface layer through a diffusion-driven process, after which ligands can be transferred to the surface forming volatile species.^34−40^ The diffusion-driven nature of the fluorination step, as well as the modified surface layer acting as a diffusion barrier, causes the modification step to soft-saturate, as seen for both thermal and plasma isotropic ALE.^36−38,40^ Despite the soft saturation, a high level of etch control is still possible, and this behavior is exhibited in many ALE processes.^32−39^ In advanced technology nodes, it is important to ensure that the surface quality is as high as possible. To this end, the benefits of both ALD and ALE can be applied in combined processing, that is, performing ALE directly after ALD. Such applications have been discussed in previous work;^23^ however, demonstrations are rare in the literature.

In this work, we describe the smoothing via ALD and ALE using finite difference simulations to generate a model for smoothing, giving insight into the underlying mechanisms. These simulation results were validated in experimental ALD and ALE studies, involving Al_2_O_3_ ALD from TMA/O_2_ plasma and isotropic Al_2_O_3_ ALE from TMA/SF_6_ plasma. The effect of surface fluorination on the rate of smoothing is discussed and incorporated into the model to explain the enhanced smoothing seen for ALE. A stack of alternating ZnO/Al_2_O_3_ was deposited and etched to highlight the benefits of combined processing, showing that the smoothest thin film is made possible by combined ALD/ALE processing. Finally, from this demonstration, further strategies for employing combined ALD and ALE to smoothen surfaces for various applications are discussed.

## 2 Experimental Methods

In order to measure the effect of ALD on the surface roughness, a rough starting surface is required, which is created by depositing polycrystalline ZnO on a smooth Si surface using ALD. ZnO was deposited using a thermal process in an Oxford Instruments OpAL reactor, using 60 ms diethylzinc, 5 s purge, 60 ms H_2_O, and 10 s purge steps.^41^ Depositions were performed at 200 °C, after which the samples were annealed at 450 °C for 10 min in N_2_, such that the films are fully crystallized. The samples were then exposed to an O_2_ plasma for 10 min. Films with two different thicknesses of ZnO were deposited, that is, 47 nm by performing 300 cycles and 98 nm using 600 cycles. The thicker film has larger crystal grains and a higher surface roughness. Al_2_O_3_ films of different thicknesses were deposited on both ZnO films, as well as on a smooth Si substrate as a control. Al_2_O_3_ was deposited using a plasma ALD process in an Oxford Instruments FlexAL reactor, with a substrate table temperature of 150 °C. The ALD cycle consisted of 40 ms trimethylaluminum (TMA), 3 s purge, 3 s O_2_ plasma at 200 W and 25 mTorr, and 3 s purge steps. Films with 11 different thicknesses of Al_2_O_3_ were deposited, ranging from 1 nm to 173 nm.

The data set to measure the effect of isotropic ALE on the surface roughness was obtained using 41 nm Al_2_O_3_ films deposited on 52 nm of ZnO (300 cycles). The initial films have a roughness, caused by the roughness of the underlying ZnO film and the inherent roughness of the Al_2_O_3_ film, as is discussed in Supporting Information (SI) Section S.A. The isotropic ALE process utilizes SF_6_ plasma and TMA and was performed in an Oxford Instruments FlexAL reactor, with the substrate table set to 300 °C.^39^ The Al_2_O_3_ ALE recipe used a 40 s SF_6_ plasma at 300 W and 50 mTorr by supplying 50 sccm Ar and 100 sccm SF_6_. TMA is dosed for 50 ms with the automatic pressure control (APC) valve set to 200 mTorr and is held for 2 s at 200 mTorr before the next TMA dose; the dose and the hold steps are repeated 10 times each cycle. The reactor is purged in between each half-reaction for 10 s with 100 sccm Ar.

For investigating the potential benefits of combined ALD and ALE processing, a stack of alternating ZnO and Al_2_O_3_ layers was deposited. The three ZnO layers were 55 nm thick, and after each ZnO deposition, the stack was annealed for 10 min at 450 °C in an N_2_ environment. An Al_2_O_3_ layer was deposited between each ZnO layer. The first Al_2_O_3_ layer was prepared by performing 35 nm of ALD, followed by 20 nm of ALE. The SF_6_ plasma step was adjusted such that a 10 s SF_6_ plasma at 100 W and 100 mTorr with 150 sccm Ar and 50 sccm SF_6_ was used. The second Al_2_O_3_ layer was deposited with ALD to a thickness of 15 nm, and the third Al_2_O_3_ layer was deposited to 35 nm. Multiple 4 × 4 cm Si samples were loaded at the beginning, such that after each ALD/ALE process, one sample could be removed to monitor the roughness and thickness of each layer using atomic force microscopy (AFM) and spectroscopic ellipsometry. The sample exposed to all process steps was analyzed using a transmission electron microscope to investigate the interfaces between the deposited layers.

AFM measurements were performed on the Dimension Icon AFM manufactured by Bruker, operated in PeakForce with the ScanAsyst mode, using a PeakForce-Air tip. The scan frequency was set to 1 Hz. Spectroscopic ellipsometry (SE) measurements were performed on an ex situ J.A. Woollam M-2000 variable angle spectroscopic ellipsometer at the angles of 65°, 70°, and 75°. The data was acquired between 1.24 and 6.5 eV, with 512 wavelength steps and an acquisition time of 5 s. In situ SE measurements were also carried out using a J.A. Woollam M-2000 spectrometer with the angle fixed at 70° and window effects enabled.

Transmission electron microscopy (TEM) and scanning TEM (STEM) studies were performed using a JEOL ARM 200F TEM, probe corrected, equipped with a 100 mm^2^ Centurio SDD EDX detector, operated at 200 kV. The studied stacks were deposited on Si coupons and then cut by a focused ion beam to produce a cross section. Both annular bright-field (ABF) and high-angle annular dark-field (HAADF) images of the cross sections were taken using STEM. In total, 20 STEM images were recorded and used to determine roughness as outlined in SI Section S.B. Energy-dispersive X-ray (EDX) mapping was also performed on the cross section to examine the elemental composition.

## 3 Simulation Methods

The etching/deposition of a thin amorphous film is represented in a model by describing the height *h_i,j_* of the film at discretized positions (*x*, *y*). For ALD and ALE processes, the change in thickness during each cycle is given by the growth per cycle (GPC) and etch per cycle (EPC), respectively. The model operates as a function of the deposited/etched thickness (τ), which is defined by the GPC/EPC, multiplied by the number of cycles. In the model, it is assumed that each individual ALD or ALE cycle deposits or etches a uniform layer, which differs from experimental observations where a sub-monolayer amount of the material is deposited or etched per cycle. In practice, the deposition/etching of sub-monolayers adds up to a uniform layer, and therefore, by executing the model over multiple cycles, a reasonable approximation for the advancing deposition or etch front is achieved.

The model is based on a square grid, with size *N*^2^ and length *L*. This grid matches the format of experimental data from AFM measurements, meaning that AFM data can be used as the initial condition of the model (τ = 0).

The roughness is quantified using the RMS roughness value and the power spectral density (PSD).^42^ The PSD is defined as
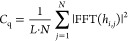
1with *h_i,j_* the height of the surface and FFT() the fast Fourier transform. The PSD has unit m^3^ and is a function of the wavenumber (*q*), which has unit m^–1^. For a rough surface, the PSD gives the prevalence of roughness at a certain spatial frequency. For example, a rough surface with laterally large features has a PSD contribution at low wavenumbers. A surface with small and sharp features has a PSD contribution at high wavenumbers. For most surfaces (and all surfaces investigated in this work), the PSD is constant for low wavenumbers and decreases to zero beyond a certain wavenumber, and this wavenumber is defined as the reciprocal of the correlation length.^43^ AFM measurements always contain a certain amount of electronic noise, which is especially significant for high wavenumbers. This noise level is corrected for, as explained in SI Section S.C.

The model is inspired by the work of Sethian et al.,^44^ in which a model was developed that simulates etch and deposition processes as a propagating front. The propagation is in the direction locally normal to the surface, and given by *F·*τ, with *F* the dimensionless rate of propagation. For an idealized ALD or ALE process, the propagation each cycle is equal everywhere, and as such, *F* = 1. Therefore, the thickness deposited or etched at each surface point is equal to τ. Since in this case the propagation is uniform, it is referred to as “uniform front propagation” (UFP). The propagation of the front in the vertical direction is given by the differential equation
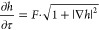
2which is the equation as given in the work by Alasaarela et al. when *F* = 1.^31^ The derivation of eq 2 can be found in SI Section S.D. Eq 2 is solved with the finite differences method using the upwind solution scheme.^45^ τ is discretized with steps of Δτ = 0.1 nm, meaning that the model results in a series of surfaces, at an interval of 0.1 nm of deposition or etching. The resulting surfaces are compared to experimental AFM data using the metrics RMS roughness and the PSD.

Experimentally isotropic ALD and ALE processes are considered without substrate biasing (and a relatively high pressure for ALE) during their respective plasma half-cycles. These conditions correspond to radical-driven plasmas with minimal incident ion energy. The model therefore describes the roughness evolution for ALD and ALE of amorphous materials using processes that are not significantly determined by plasma–surface interactions.

**Figure 1:**
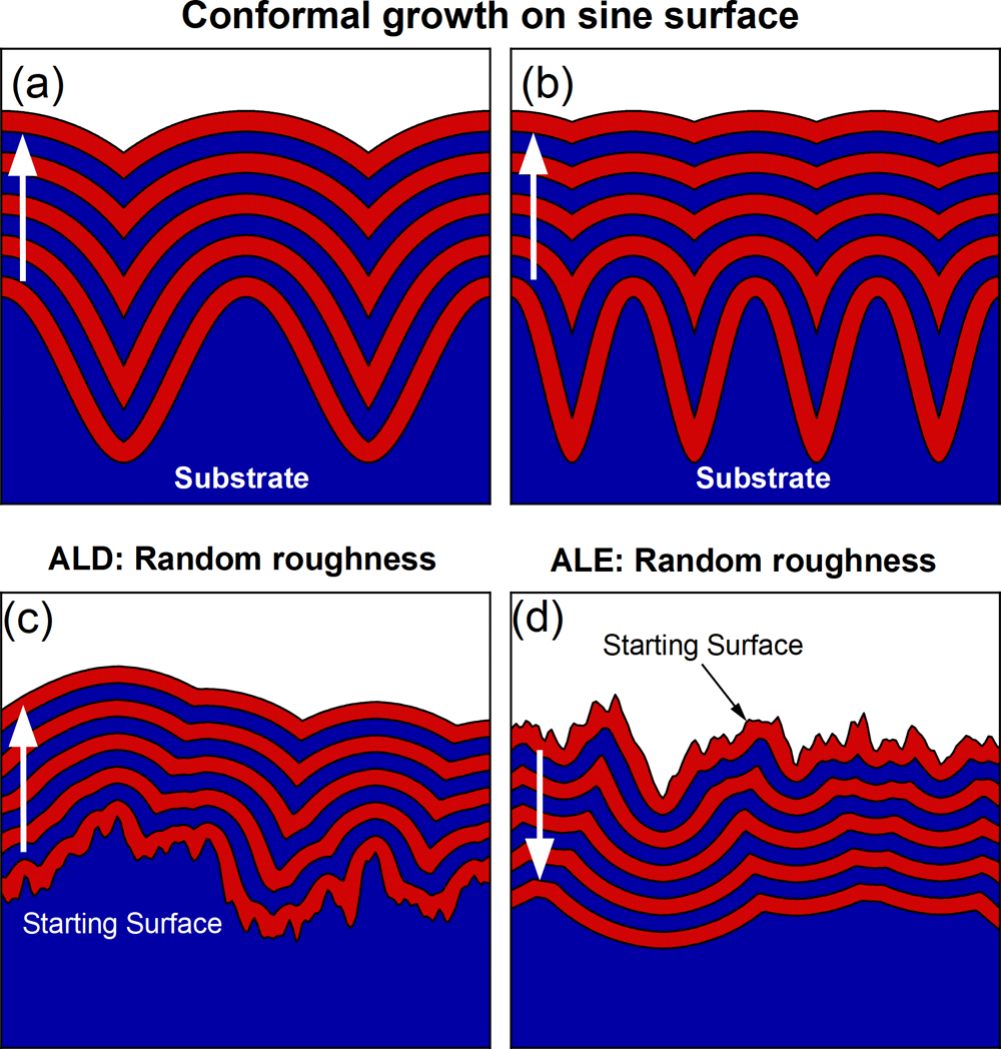
Illustration of the UFP model in 1D where the white arrow indicates the direction of propagation for growth and etch for nine steps of the model. The starting surface is shown in gray. Each step of the model indicates one ALD or ALE cycle. (a,b) Conformal growth on sine-shaped surfaces with different spatial frequencies. (c) ALD on a randomly rough surface. (d) ALE on a randomly rough surface. In both (c,d), it can be seen that the roughness is reduced, with small-scale features being removed from the film.

To illustrate the model, several test cases were explored in one dimension. The first case is ALD on a sine-shaped surface, as shown in Figure 1a,b. Conformal deposition leads to the filling of the valley from the sidewalls inward, creating a sharp valley. Figure 1a,b clearly shows that conformal deposition leads to a reduction in surface roughness and that a surface with high spatial frequencies is smoothed faster. ALD and ALE are also modeled on randomly rough surfaces in Figure 1c,d, respectively. Conformal deposition leads to the formation of broad peaks and sharp valleys, and the small-scale features of the roughness are eliminated. Similar results are observed for the ALE model but in the opposite direction.

## 4 Results and Discussion

Two different processes were investigated for their ability to smoothen surfaces: plasma-enhanced ALD of Al_2_O_3_ using TMA and O_2_ plasma and isotropic plasma ALE of Al_2_O_3_ using TMA and SF_6_ plasma.

### 4.1 ALD of Al_2_O_3_

**Figure 2:**
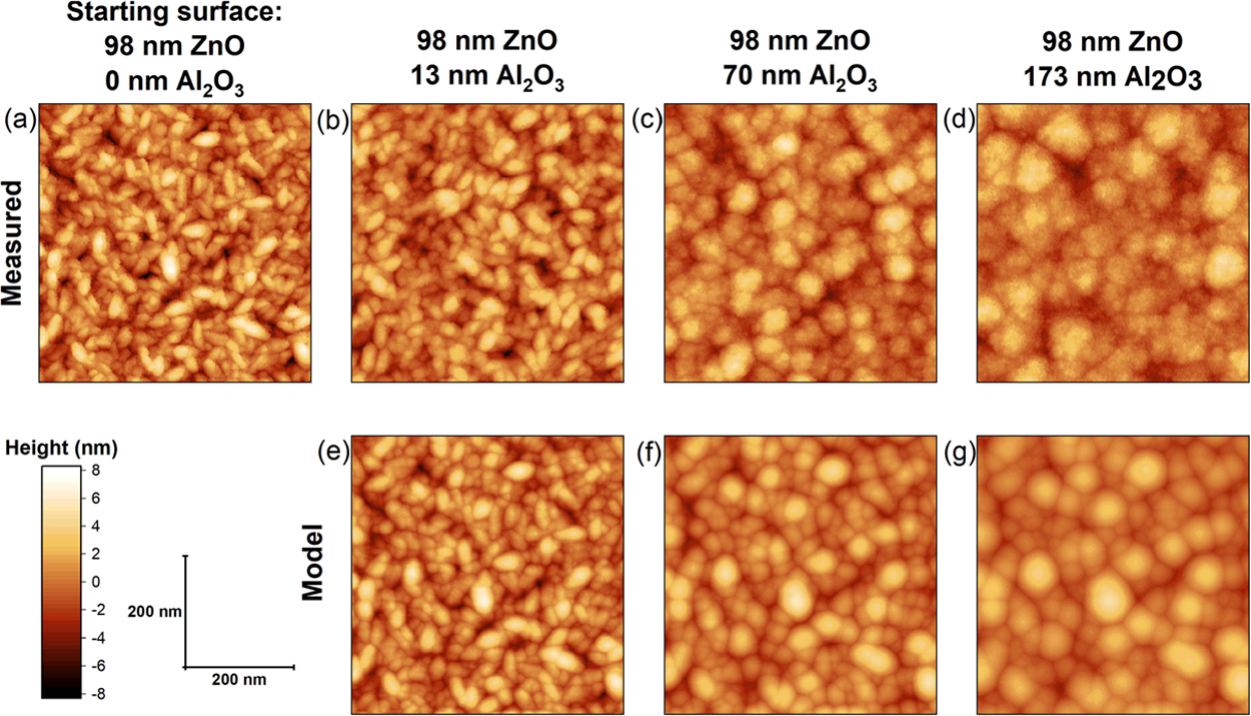
(a) AFM heightmap of the 98 nm ZnO sample, which was used as an input for the model. (b–d) AFM heightmaps measured after Al_2_O_3_ ALD of different thicknesses. (e–g) Heightmaps calculated using the UFP model for films of the same thickness as in (b–d).

In Figure 2, AFM heightmaps for different thicknesses of Al_2_O_3_ deposited on the 98 nm ZnO sample are shown. The AFM heightmaps in Figure 2a–d are compared to heightmaps calculated by the UFP model in Figure 2e–g for the same Al_2_O_3_ film thicknesses. Looking first at the experimental data, we see that for an Al_2_O_3_ thickness of 13 nm, the individual crystals of ZnO are still visible but enlarged by the deposition. After 70 nm of Al_2_O_3_ deposition, the features have grown larger, no longer resembling the ZnO crystals. Peaks have broadened, while the valleys are sharp and narrow, a trend which is continued for the 173 nm thick film. For each of the thicknesses, the surfaces measured using the AFM are visually very similar to the surfaces generated using the UFP model.

**Figure 3:**
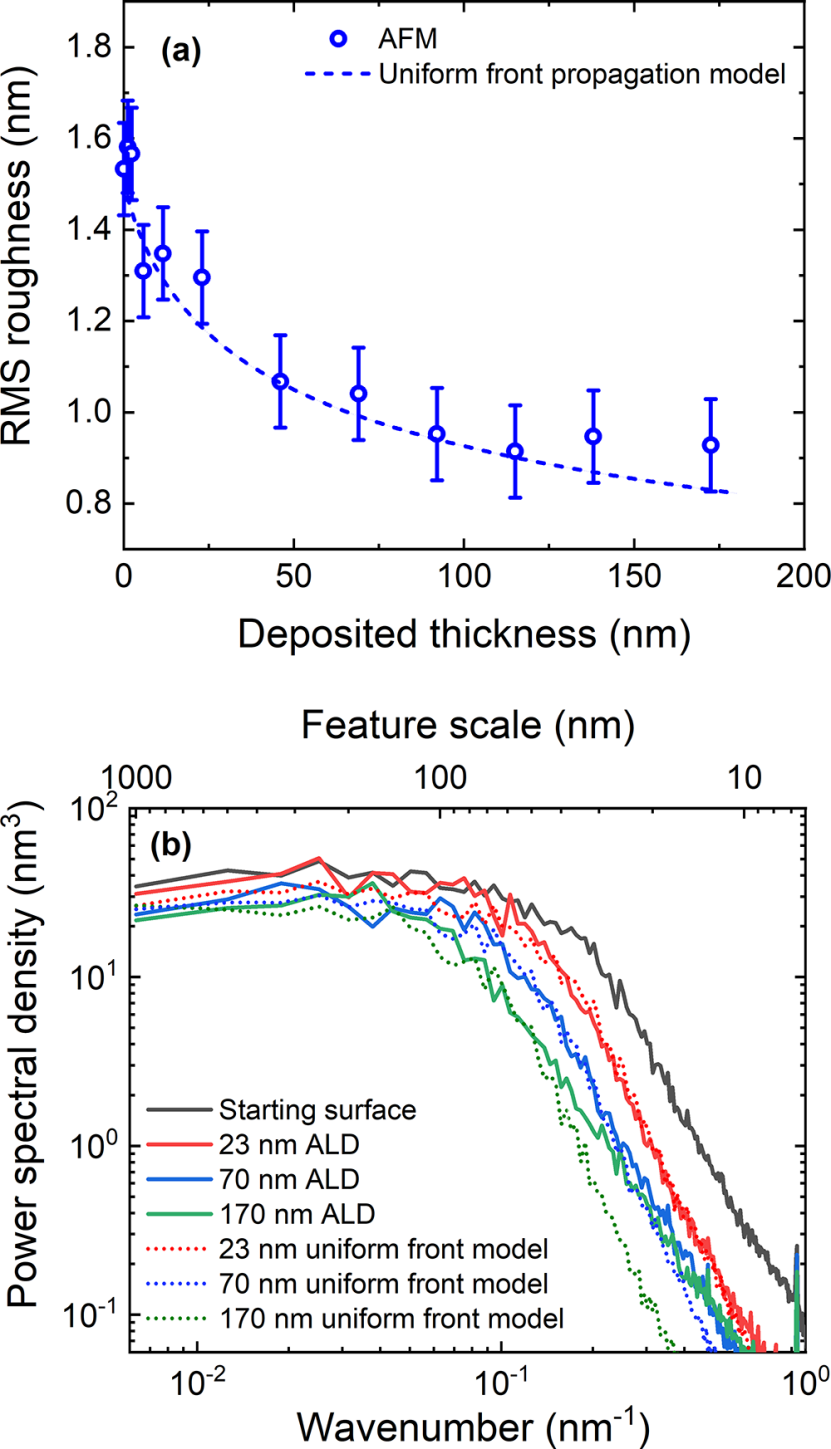
(a) Comparison of the RMS roughness as a function of deposited Al_2_O_3_ thickness on the 47 nm ZnO sample from experimental and model data. (b) PSD from the AFM data compared to PSD from the model for different deposition thicknesses. The measured AFM data deviates in the high-wavenumber range due to contribution of the inherent roughness (see Section S.A). The PSD is corrected for the contribution of the noise using a constant value for the noise (see Section S.C).

From Figure 2, it is observed that small-scale features are smoothed out first, for instance the small gaps between the ZnO crystal grains, while features much larger than the thickness of the deposited Al_2_O_3_ persist. In Figure 3a, the RMS roughness is plotted as a function of the deposited film thickness, with the experimentally observed reduction in roughness being described very well by the UFP model. As a reference measurement, ALD on a smooth Si substrate was found to lead to a slight increase in roughness of 0.2 nm RMS after 1500 cycles of ALD (SI Section S.A Figure S.1), which is due to the inherent roughness of the ALD process.^17,46,47^ The effects of the inherent roughness can also be seen in Figure 2d as the grainy features in the AFM image of the 173 nm thick Al_2_O_3_ film. For thicker films, Figure 3a shows that the reduction in roughness begins to plateau. Where this plateau in roughness occurs is likely dependent on both the roughness of the starting substrate and the inherent roughness of the ALD process. Plateauing of the RMS roughness during ALD has previously been reported for ALD of Al_2_O_3_.^17,28^ Overall, the UFP model accurately describes the smoothing behavior of ALD Al_2_O_3_, showing that most of the smoothing occurs during the initial ALD cycles and that smoothing occurs by conformal filling of features by ALD.

In Figure 3b, the 1D PSD of experimental and model data are compared. The PSDs are corrected for the electronic noise component, as explained in SI Section S.C. A significant reduction in PSD is observed at high wavenumbers, indicating that the small-scale roughness is reduced fastest by ALD. For thicker films, the reduction in the high wavenumber range is more significant. At high wavenumbers, the reduction in PSD saturates, with no further significant decrease in PSD observed between a 70 and 170 nm deposited Al_2_O_3_ film. This plateau in small-scale roughness reduction is also likely due to the initial substrate roughness and the inherent roughness of the ALD process as is explained in SI S.A. However, the high-wavenumber region is the most affected by electronic noise which could also contribute toward the plateau in PSD reduction. The PSD in the low-wavenumber range (corresponding to large-scale features) decreases only slightly. Both RMS roughness and PSD are accurately described by the UFP model, highlighting its utility for examining the mechanism by which smoothing occurs for ALD.

### 4.2 ALE of Al_2_O_3_ Using TMA and SF_6_ Plasma

**Figure 4:**
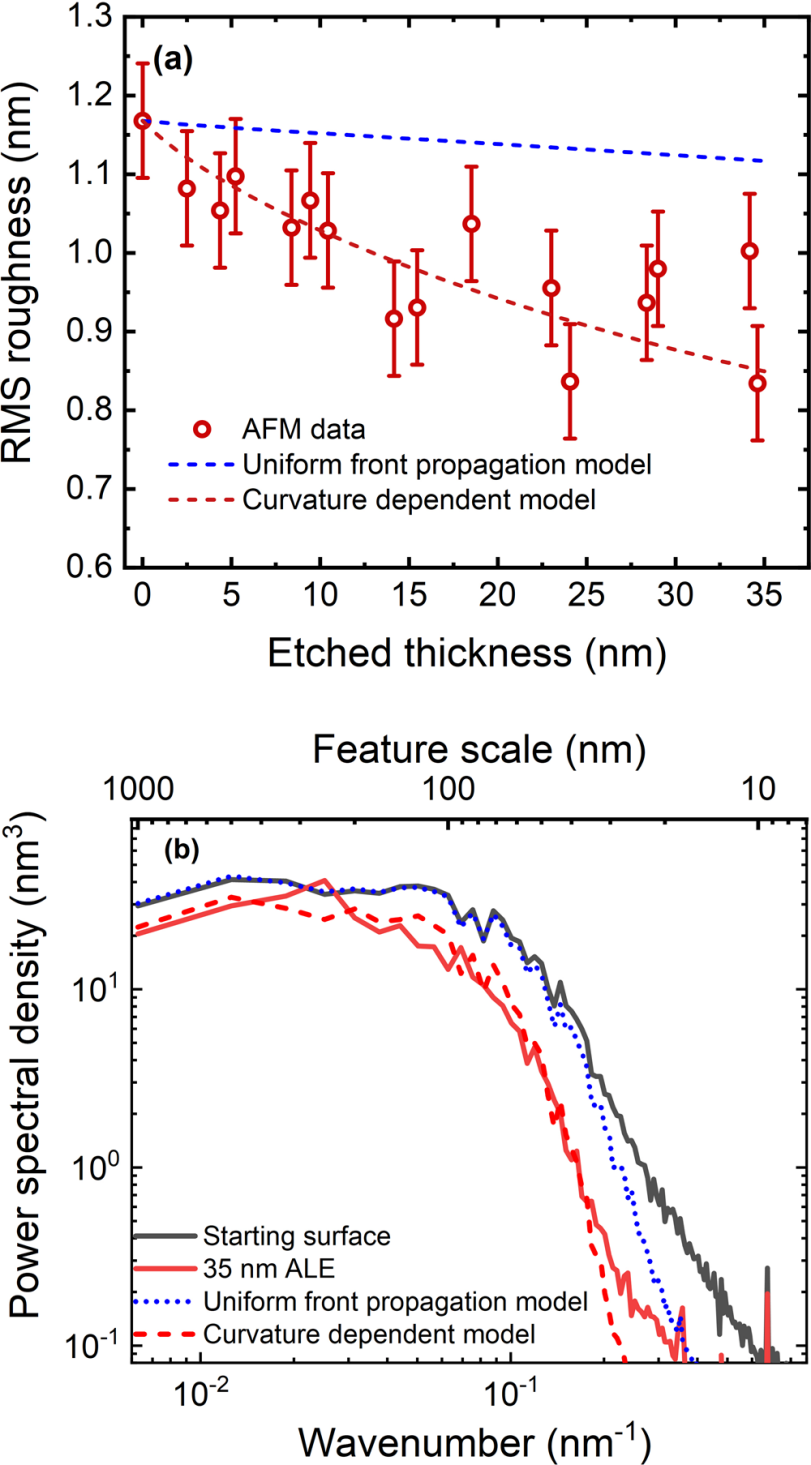
(a) RMS roughness after ALE using TMA and SF_6_ plasma on 41 nm Al_2_O_3_ on 47 nm of ZnO. The curvature-dependent propagation (CDP) model is fitted using ε *=* 1.5 × 10^–9^ m. (b) The PSD of 41 nm Al_2_O_3_ ALD on 47 nm ZnO, the PSD after 35 nm of ALE using TMA and SF_6_ plasma, and model data of 35 nm of ALE using ε = 1.5 × 10^–9^ m for the CDP model and ε = 0 m for the UFP model. The deviation between the model and experimental data for high wavenumbers is caused by the measurement noise.

To test the effect of ALE using TMA and SF_6_ plasma on the surface roughness, a rough sample was made by depositing 41 nm of Al_2_O_3_ on 52 nm of ZnO, which has a final RMS roughness of 1.17 ± 0.07 nm. The RMS roughness as a function of etched thickness is shown in Figure 4a. After 150 cycles of ALE, 35 nm of the material was removed, and the RMS roughness was reduced to 0.83 ± 0.07 nm. For ALE, the UFP model only predicts limited smoothing of the surface, which is seen in both the RMS roughness and PSD in Figure 4b. However, the smoothing observed in the experiments is much greater than that given by the UFP model. This suggests that in addition to smoothing by UFP, a second smoothing effect plays a role here.

The hypothesized principle leading to the faster smoothing for ALE processes involving fluorination is the dependence of fluorination on local surface curvature. As discussed in the Introduction section, the fluorination reaction soft-saturates due to its diffusion-driven nature, while the modified AlF_3_/AlF_*x*_O_*y*_ surface layer acts as a diffusion barrier.^23,38,39^ This is similar to the Deal–Grove model for the oxidation of Si for which the local curvature of the surface has been shown to affect the thickness of the oxide layer that is generated.^48−50^ Similarly, during fluorination, it can be expected that local curvature also determines the thickness of the fluorinated layer, thereby influencing how far the etch front propagates each cycle. It is anticipated that there are two main factors driving local differences in fluorination: (i) at convex regions, there is a higher supply of fluorinating species due to the wide exposure of the surface to the fluorine plasma, which based on the Deal–Grove model will produce a thicker fluorinated region.^48−50^ (ii) Convex regions (peaks) are fluorinated more easily as the volume expansion required during fluorination is not inhibited by the surrounding material, while the inverse is true for concave regions(valleys).^19,37,49,50^

**Figure 5:**
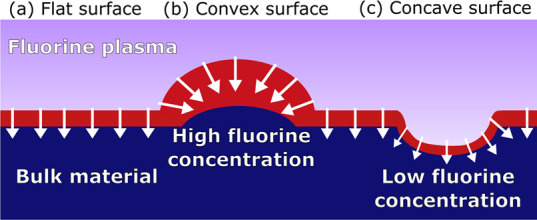
(a) Fluorination of a flat surface. Fluorine radicals adsorb on the surface and diffuse through the fluorinated layer, reaching the underlying material where it reacts to fluorinate the material. (b) On a convex surface, there is more surface area for radicals to absorb, relative to the surface area of the nonfluorinated material interface. This results in relatively faster fluorination of the material compared to a flat surface. The convex surface is also less constrained by the surrounding material and is thus able to more easily undergo the surface expansion required during fluorination. (c) On a concave suface, the opposite effects are true, leading to relatively slower fluorination.

In an adapted model that includes curvature-dependent fluorination, the dependence of the rate of front propagation *F*, on the curvature *K*(*x*, *y*) (defined in SI Section S.D), is given by *F* = 1 – ε*K*, with ε the diffusion parameter.^44^ The mean curvature on peaks is negative, resulting in faster propagation of the etch front, while the curvature is positive in valleys giving slower front propagation. The enhanced F concentration at convex areas (i.e., at peaks) and a reduced F concentration at concave areas (i.e., in valleys) is illustrated in Figure 5, which leads to peaks and valleys leveling out over multiple ALE cycles. For ALE mechanisms involving fluorination, there are thus two processes which lead to smoothing: uniform front propagation + curvature-dependent smoothing. The curvature-dependent propagation (CDP) model combines both mechanisms, for which values for ε are determined through fitting the model to the experimental data. The diffusion parameter gives the impact of diffusion on the propagation, where high values of ε denote a more significant impact of diffusion.

Applying the CDP model to datasets upon which Figure 4a is based shows that a nonzero value of ε leads to faster smoothing. The value of ε which provides the best fit of the model to the experimental data was found to be ε *=* 1.5 × 10^–9^ m. In Figure 4b, the PSD for the starting surface and the surface after 35 nm of ALE as measured by AFM are shown alongside the predictions of both models using the starting surface as an input. Comparing the experimental data to the two models shows that the CDP model matches well, with the two curves only deviating at high wavenumbers, which is likely due to noise. Similar to ALD, ALE leads to a reduction in the PSD over the whole spectrum, with the majority of smoothing occurring at high wavenumbers. This suggests that ALE acts to quickly remove the small-scale roughness on the film and would struggle to smooth features that are much larger than the total etched thickness of the film. An additional similarity to ALD is that smoothing can be limited by factors such as initial surface roughness and inherent roughening of the process, which is not clearly shown in Figure 4a. A data set illustrating this behavior for ALE is included in SI Section S.A. The smoothing via ALE can be accurately predicted with the CDP model, allowing for the modeling of AFM heightmaps for ALE. In Figure S7, both measured and modeled height maps are shown. The height maps generated using the CDP model, with ε *=* 1.5 × 10^–9^ m, match well with the height maps measured by AFM. While this model can accurately predict and model the experimental data, the hypothesis of enhanced smoothing based on the film curvature needs to be confirmed experimentally.

### 4.3 Characterization of Smoothing by Cross-Sectional TEM

**Figure 6:**
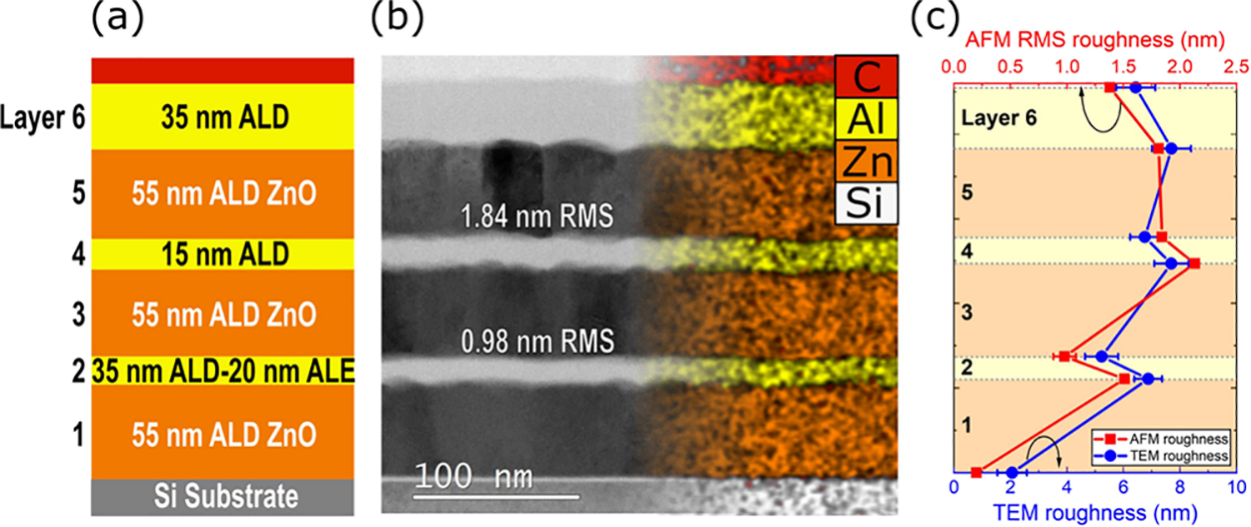
(a) Description of the ZnO/Al_2_O_3_ stack. (b) TEM image and EDX map of the stack. The interface between layers 2 and 3 is visibly smoother than between layers 4 and 5. (c) Roughness values measured by AFM and TEM analysis. Both techniques follow a similar trend in roughness and show that layer 2 is the smoothest.

To demonstrate the smoothing that can be achieved by ALD and ALE, a stack of ZnO and Al_2_O_3_ films was deposited, as shown in Figure 6a, and studied using TEM and EDX. A witness sample was removed after every ALD or ALE run in order to determine the roughness by AFM. By preparing multiple layers using ALD and ALE, we highlight that combined ALD/ALE processing offers the best results for thin and smooth films. The TEM image in Figure 6b shows that the interfaces above the light gray-colored, amorphous Al_2_O_3_ are much smoother than the surfaces above the dark-colored, polycrystalline ZnO. Layers 4 and 6 deposited using ALD, which are 15 and 35 nm thick, respectively, provide a comparison between smoothing for different deposition thicknesses. We observe that 15 nm ALD Al_2_O_3_ gives an RMS roughness reduction of 0.29 ± 0.07 nm with respect to the ZnO surface, whereas for the 35 nm Al_2_O_3_, there is enhanced smoothing with a reduction of 0.42 ± 0.07 nm. This difference in roughness can also be observed in the TEM image of Figure 6b, in which layer 4 has a more blurred top surface compared to layer 6.

In order to compare ALD and ALD + ALE, layer 2 is also 15 nm thick but is made by first depositing 35 nm and then etching back 20 nm. Measurement of the roughness after the initial 35 nm ALD Al_2_O_3_ shows a reduction of 0.22 ± 0.06 nm RMS roughness, which is reduced by an additional 0.31 ± 0.11 nm after 20 nm of ALE, representing a total reduction in RMS roughness of 0.53 ± 0.10 nm. The reduction obtained after 20 nm ALE is more than observed for 35 nm ALD, highlighting that ALE results in faster smoothing than ALD.

Roughness values for the layers were also determined from the TEM images, as described in more detail in SI S.B. The TEM overestimates the roughness of the films; however, the trend in the data sets is very similar, as can be seen in Figure 6c. Both methods indicate that ALD of Al_2_O_3_ films leads to smoothing. Deposition of more materials results in a larger reduction in roughness, which is shown by layer 6 being smoother than layer 4. From the TEM and EDX images in Figure 6b, it can be deduced that the interface between layers 2 and 3, corresponding to the ALD + ALE surface, is the smoothest. This stack demonstrates that to achieve ultrathin, smooth films, it is best to first deposit a thicker film with ALD and then etch back with ALE.

### 4.4 Comparison of Used Processes

To make a comparison between our own data and reports from the literature, a common metric of how roughness evolves as a function of process cycles is required. For smoothing, this is less straightforward than for say deposition or etching where values for GPC or EPC provide easy comparison between different process conditions and literature. The metric reduction in RMS roughness per ALD cycle (nm/cycle) has been used in literature to describe the smoothing ability of a process.^17^ However, this metric has the drawback that it does not take the roughness before the processing, the inherent roughness of the ALD process, nor the difference in GPC or EPC into account, all of which can influence roughness evolution. An alternative method of quantifying smoothing that partially addresses these drawbacks is to monitor the reduction in film roughness (nm) per unit of deposited/etched thickness (nm), here referred to as the smoothing rate(nm/nm). The reduction in roughness and the deposited/etched thickness should preferably be taken when the roughness begins to saturate (see Figure 3a). Exact determination of when saturation of smoothing occurs can be difficult, but the analysis still provides some general trends for the rate at which smoothing proceeds. An overview of smoothing rates observed in our work and notable examples from literature is shown in Table 1. A similar analysis is performed for the ZnO/Al_2_O_3_ stack and can be found in SI Section S.E.

**Table 1:** Comparison of the Smoothing Rate between This Work and the Literature for ALD and ALE of Al_2_O_3_ Films

	initial roughness (nm)	final roughness (nm)	deposited/etched thickness (nm)	smoothing rate (×10^–3^ nm/nm)	reference
ALD Al_2_O_3_ on 47 nm ZnO	1.53	0.91	115	5.8	this work
ALD Al_2_O_3_ on 98 nm ZnO	2.00	1.45	115	5.2	this work
ALD Al_2_O_3_	3.30	1.50	370	4.9	Myers et al.^28^
ALE Al_2_O_3_ on 41 nm ZnO	1.17	0.83	35	9.8	this work
ALE Al_2_O_3_	0.63	0.37	10	26	Zywotko et al.^34^

For ALD experiments performed in Section 4.1, Table 1 shows that a comparable rate of smoothing is obtained for both ALD films which have different starting surfaces. Furthermore, in Myers et al., the initial surface is smoothed with a rate of 4.9 × 10^–3^ nm/nm,^28^ which is comparable to the smoothing observed in this work. For our ALE experiments described in Section 4.2, a smoothing rate of 9.8 × 10^–3^ nm/nm was obtained. The higher rate of smoothing from ALE is attributed to the diffusion-driven nature of the SF_6_ modification half-cycle. Zywotko et al. used a comparable thermal ALE process, for which an initial surface roughness of 0.625 nm was smoothed with a rate of 2.6 × 10^–2^ nm/nm.^34^

### 4.5 Application of ALD and ALE for Smoothing of Surfaces

A key question is whether ALD and ALE processes can be used in the fabrication of nanoelectronics and photonics to reduce the detrimental effects of roughness on device performance. Already ALD has been adopted in a wide variety of applications, such that the smoothing effect is already present in many process flows. However, for applications where sub-10 nm films are required, such as nanoelectronics, an approach relying solely on ALD will be ineffective for achieving a smooth surface. This has been observed in our experimental data, literature, and the UFP model results, which indicate that smoothing via ALD requires relatively thick films of >20 nm.^17,28^ Conversely, ALE provided much faster smoothing of the films, both in literature and our work, but employing solely ALE for surface smoothing appears at first sight not a solution, since that would require the partial removal of a functional layer. In practice, it is therefore especially interesting to consider combinations of ALD and ALE, with most of the smoothing occurring during ALE. A similar concept as outlined here has been discussed recently, where anisotropic quasi-ALE and ALD were used to improve self-aligned contacts and shrink patterns.^51^ The combination of ALD and ALE leads to a process that shows similarities to chemical–mechanical polishing (CMP), where first a material is applied to the surface, which is then subsequently removed, resulting in a thinner film with a smooth planar surface. While CMP is used extensively to create smooth surfaces for flat substrates, CMP cannot be used for smoothing of 3D structures, such as sidewalls of trenches or around nanowires. Due to their isotropic nature, ALD and ALE can be used to smooth 3D structures and perform a similar function as CMP for advanced device structures. An additional benefit is that ALD and ALE are dry processes, meaning that integration into current high volume manufacturing process flows will be easier.

**Figure 7:**
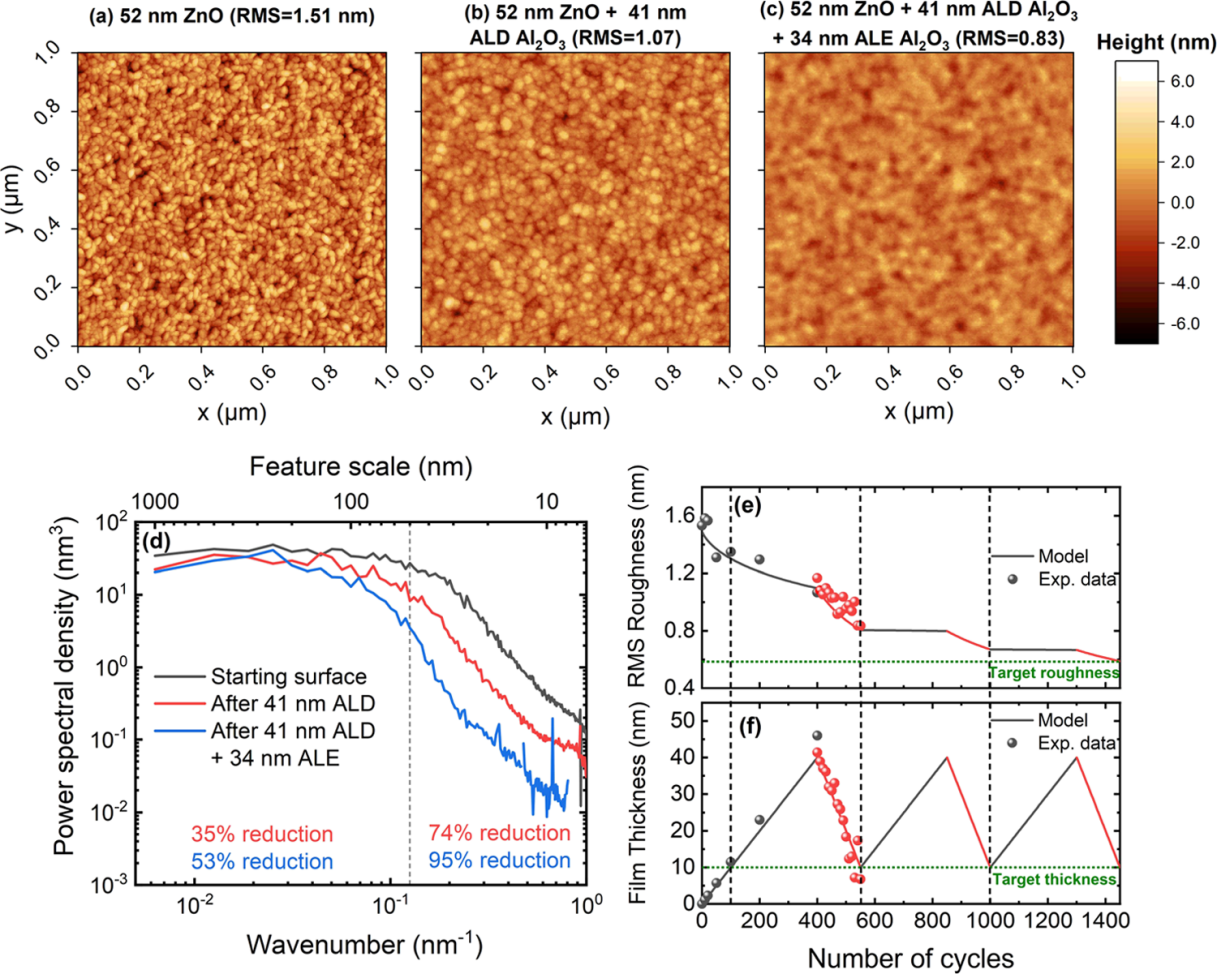
AFM height maps of samples prepared by (a) 52 nm ZnO, (b) 52 nm ZnO and 41 nm of Al_2_O_3_ deposited using ALD, and (c) 52 nm ZnO and 41 nm of Al_2_O_3_ deposited using ALD followed by 34 nm of Al_2_O_3_ etching using ALE. (d) The PSDs of samples (a–c), where the vertical dashed line indicates 50 nm. Rough features below 50 nm (wavenumbers above 2 × 10^–1^ nm^–1^) are smoothed significantly more than larger features. Film (e) roughness and (f) thickness as a function of ALD + ALE cycles plotted alongside model predictions over multiple ALD + ALE supercycles.

The ALD + ALE approach is further illustrated in Figure 7, by both model predictions and experimental data, where a rough surface is capped with a thin <10 nm film to smooth the surface. Here, the starting substrate is a 52 nm ZnO film deposited via ALD and as such has a rough surface, with an RMS roughness of 1.51 nm. The first approach is to employ only ALD to deposit a 10 nm Al_2_O_3_ film; however, this results in only a minor reduction in surface roughness from 1.51 to 1.30 nm, as is predicted by the model. A second, and perhaps better, approach is to first deposit a thicker film, which can then be etched back with ALE. For this 41 nm Al_2_O_3_ is deposited, resulting in a reduction in RMS roughness to 1.07 nm (see Figure 7b). Deposition of this thick layer provides the opportunity to perform ALE, which subsequently removed 34 nm of Al_2_O_3_, resulting in a final film thickness of 7 nm. The final surface roughness of 0.83 nm (Figure 7c) is a significant improvement on the 1.30 nm RMS value achieved using solely ALD.

In Figure 7d, the PSDs of the starting ZnO surface, the surface after 41 nm ALD, and the surface after 34 nm ALE are shown. The PSD decreases after both ALD and ALE, mostly for the high wavenumbers. To quantify this change in the PSD, the PSDs are split into two regimes: features laterally larger than 50 nm and features smaller than 50 nm. The combination of ALD and ALE leads to a 53% reduction in the PSD larger than 50 nm but a 95% reduction in the PSD for features smaller than 50 nm, again illustrating that ALD and ALE smoothing affects mostly small-scale roughness. The distinction between small- and large-scale roughness is relevant as the precise influence of the roughness on device physics depends on the spatial frequency of the roughness (determined from PSD data), as well as on the vertical distribution (RMS roughness). For example, the leakage current of a capacitor is dictated largely by the sharpness of protrusions at rough interfaces.^3,10,52^ For the scattering of light, for example, a waveguide for photonics, the intensity loss is dependent on the RMS slope, which is defined as the standard deviation of the slope of the surface.^53^ For these examples, the effect of surface roughness can be calculated from the PSD.^3,31^

How the film roughness and thickness evolve during the ALD and ALE processing can be seen in Figure 7e,f, respectively. Here, the experimental data is plotted alongside the prediction of the UFP model for ALD and the UFP + CD model for ALE, showing that both roughness and thickness are accurately predicted by the respective models. Additionally, while there is only experimental data for one supercycle, the model suggests that further reduction in film roughness can be achieved when the process is run for multiple ALD + ALE supercycles. The model predicts that the smoothing via ALD becomes minimal after the first supercycle (similar to the plateauing behavior observed earlier); however, there is still significant smoothing via ALE. After the three modeled ALD + ALE supercycles, the roughness is reduced to 0.59 nm, as compared to 0.83 nm for just one supercycle.

**Figure 8:**
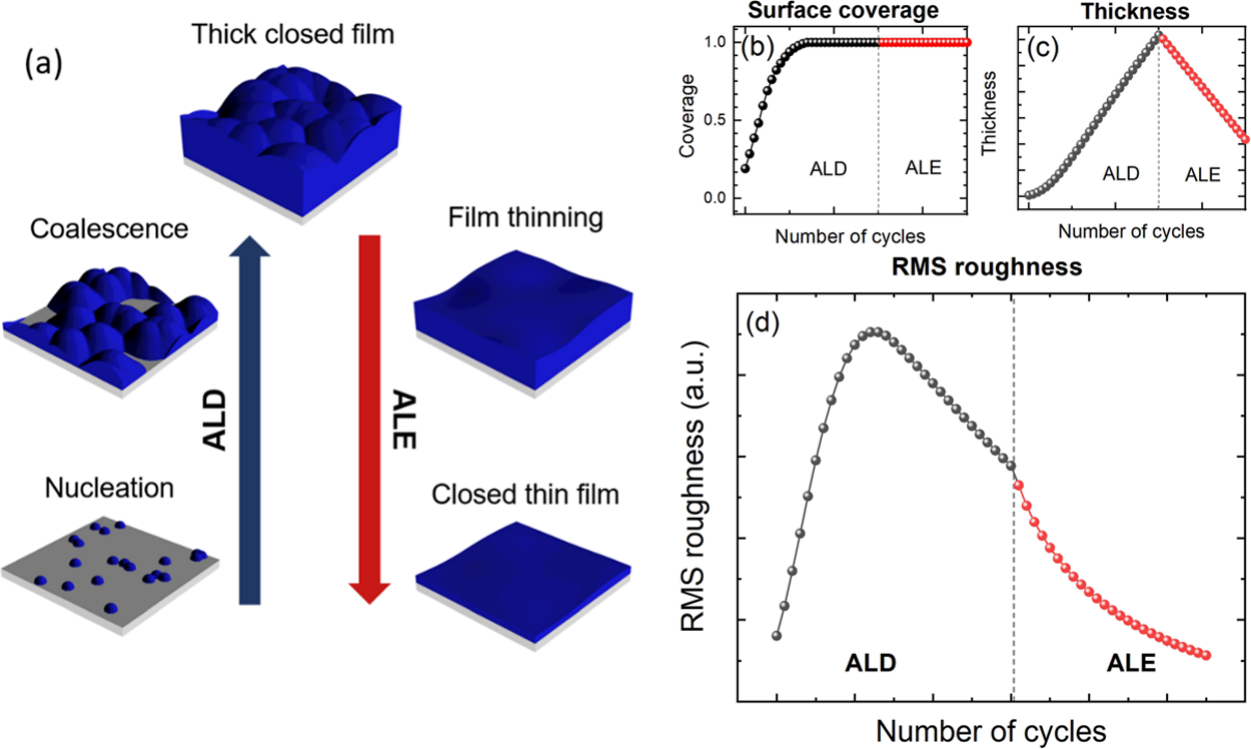
(a) Possible method to create thin and closed films by first depositing a thick film, to ensure film closure, and then to use ALE to reduce the film thickness. This figure was produced using the UFP and CDP models, with 20 randomly placed hemispherical nucleation sites of 0.4 nm initial radius. (b) Surface coverage, (c) film thickness, and (d) RMS roughness as a function of the number of cycles. The roughness peaks during island coalescence and decreases afterward for ALD, after which ALE reduces the RMS roughness even further.

A combination of ALD and isotropic ALE can also be applied for the deposition of ultrathin closed films on 3D structures, as highlighted by George et al.^23,54^ For some materials, ALD initiates by formation of islands, where a closed film is only achieved once the sufficient material has been deposited such that the islands coalesce. Creating a thin and closed film of such a material using deposition alone might not be possible depending on the number of nucleation sites. One potential route to achieving a thin film is by first performing ALD, followed by etch back using ALE. The thickness of the film can be reduced while also decreasing the roughness of the film, resulting in a thin and closed film. To investigate this application, the model was modified to use a set of hemispheres on a flat substrate as a starting surface, where deposition is only allowed to occur on the hemispheres as shown in Figure 8. For ALD, there is an initial increase in RMS roughness as the islands coalesce, but the roughness starts to decrease once full coverage is achieved. However, if the final film is required to be only a few nanometers thick, ALE could be used to reduce the film thickness and roughness while still maintaining a closed layer.

## 5 Conclusions

The fact that ALD and ALE can lead to smoothing has previously been reported, but investigation of the underlying principles has been less thoroughly explored. In this work, the smoothing abilities of ALD and ALE processes on Al_2_O_3_ films were investigated. From the experimental and modeling results presented in this work, the following general conclusions can be drawn:

- It has been hypothesized that smoothing by ALD of amorphous materials is due to the conformal nature of the deposition. Comparison of the experimental data to the UFP model shows that ALD smoothing can be described as a front propagating uniformly and perpendicular to the surface at all points.
- Smoothing by ALD requires relatively thick films due to comparatively low smoothing rates which eventually plateau. The RMS value at which roughness plateaus is based on the intrinsic roughness of the process and starting substrate roughness.
- For Al_2_O_3_ ALE from TMA/SF_6_ plasma, an additional mechanism contributes to the smoothing such that a higher rate of smoothing is obtained as compared to ALD. The hypothesis for the enhanced smoothing observed in this work is that the fluorination depends on the local curvature of the surface, corresponding to faster etching of peaks as compared to valleys. ALE processes for other materials which involve fluorination are also likely to lead to smoothing.
- The analysis of the data in terms of power spectral densities reveals that small-scale roughness is smoothed out first, while removal of large-scale roughness requires films much thicker than the scale of roughness which may be beyond the application space of ALD and ALE.
- To fabricate thin yet smooth films, an approach combining ALD and ALE can be considered. To make use of the enhanced smoothing of ALE, a strategy is to deposit a thicker film and then etch it back with ALE. Repetition of ALD and ALE in a supercycle recipe allows for meeting demanding roughness targets.

The insights presented in this work can be extended beyond the processes investigated to ALD or ALE of other amorphous materials, and for ALE, different etch chemistries can also be explored. Future studies could also focus on using combined ALD + ALE processing to improve the performance of a range of devices where smooth interfaces are vital, such as in FinFET/GAAFET, MIM capacitors, or waveguides. In the push toward sub-nanometer technology nodes, being able to accurately control film thickness while maintaining, or improving, interface quality is essential.

## Supporting Information Available

Details on inherent roughness of ALD and ALE processes, roughness determination from TEM images, PSD noise correction, model details, and smoothing rate data for the ZnO/Al_2_O_3_ stack (PDF)

## References

[ref1] Wang X.; Brown A. R.; Cheng B.; Asenov A. Statistical Variability and Reliability in Nanoscale FinFETs. In Technical Digest - International Electron Devices Meeting, IEDM, 2011; pp 103–106.

[ref2] Lau W. S.; Yu D. Q.; Wang X.; Wong H.; Xu Y. Confirmation of the Surface Smoothing Effect of Atomic Layer Deposition and the Physical Mechanism Responsible for Such an Effect. In China Semiconductor Technology International Conference 2016, CSTIC 2016, 2016; pp 3–5.

[ref3] Zhao Y.-P.; Wang G.-C.; Lu T.-M.; Palasantzas G.; de Hosson J. T. M. Surface-Roughness Effect on Capacitance and Leakage Current of an Insulating Film. Phys. Rev. B 1999, 60, 9157–9164. 10.1103/PhysRevB.60.9157.

[ref4] Yeh M. S.; Luo G. L.; Hou F. J.; Sung P. J.; Wang C. J.; Su C. J.; Wu C. T.; Huang Y. C.; Hong T. C.; Chen B. Y.; Chen K. M.; Wu Y. C.; Izawa M.; Miura M.; Morimoto M.; Ishimura H.; Lee Y. J.; Wu W. F.; Yeh W. K. Ge FinFET CMOS Inverters with Improved Channel Surface Roughness by Using In-Situ ALD Digital O3 Treatment. IEEE J. Electron Devices Soc. 2018, 6, 1232–1237. 10.1109/JEDS.2018.2878929.

[ref5] Tienda-Luna I. M.; Ruiz F. G.; Godoy A.; Biel B.; Gámiz F. Surface Roughness Scattering Model for Arbitrarily Oriented Silicon Nanowires. J. Appl. Phys. 2011, 110, 084514 10.1063/1.3656026.

[ref6] Espineira G.; Nagy D.; Indalecio G.; Garcia-Loureiro A. J.; Kalna K.; Seoane N. Impact of Gate Edge Roughness Variability on FinFET and Gate-All-around Nanowire FET. IEEE Electron Device Lett. 2019, 40, 510–513. 10.1109/LED.2019.2900494.

[ref7] Patel K.; Liu T. J. K.; Spanos C. J. Gate Line Edge Roughness Model for Estimation of FinFET Performance Variability. IEEE Trans. Electron Devices 2009, 56, 3055–3063. 10.1109/TED.2009.2032605.

[ref8] Lin Y.; Alit Apriyana A. A.; Yu Li H.; Tan C. S. Three-Dimensional Capacitor Embedded in Fully Cu-Filled Through-Silicon Via and Its Thermo-Mechanical Reliability for Power Delivery Applications. In 2020 IEEE 70th Electronic Components and Technology Conference (ECTC); IEEE, 2020; vol. 2020, pp 393–398.

[ref9] Son J. Y.; Maeng W. J.; Kim W. H.; Shin Y. H.; Kim H. Interface Roughness Effect between Gate Oxide and Metal Gate on Dielectric Property. Thin Solid Films 2009, 517, 3892–3895. 10.1016/j.tsf.2009.01.117.

[ref10] Gaillard N.; Pinzelli L.; Gros-Jean M.; Bsiesy A. In Situ Electric Field Simulation in Metal/Insulator/Metal Capacitors. Appl. Phys. Lett. 2006, 89, 133506 10.1063/1.2357891.

[ref11] Poulton C. G.; Koos C.; Fujii M.; Pfrang A.; Schimmel T.; Leuthold J.; Freude W. Radiation Modes and Roughness Loss in High Index-Contrast Waveguides. IEEE J. Sel. Top. Quantum Electron. 2006, 12, 1306–1321. 10.1109/JSTQE.2006.881648.

[ref12] Morichetti F.; Canciamilla A.; Melloni A. Statistics of Backscattering in Optical Waveguides. Opt. Lett. 2010, 35, 1777 10.1364/ol.35.001777. 20517413

[ref13] Nilsen O.; Karlsen O. B.; Kjekshus A.; Fjellvåg H. Simulation of Growth Dynamics in Atomic Layer Deposition. Part II. Polycrystalline Films from Cubic Crystallites. Thin Solid Films 2007, 515, 4538–4549. 10.1016/j.tsf.2006.11.024.

[ref14] Barabási A.-L.; Stanley H. E. Fractal Concepts in Surface Growth; Cambridge University Press, 1995.

[ref15] Ono K.; Nakazaki N.; Tsuda H.; Takao Y.; Eriguchi K. Surface Morphology Evolution during Plasma Etching of Silicon: Roughening, Smoothing and Ripple Formation. J. Phys. D: Appl. Phys. 2017, 50, 414001 10.1088/1361-6463/aa8523.

[ref16] Shin C. Springer Series in Advanced Microelectronics 56 Variation-Aware Advanced CMOS Devices and SRAM, 56th ed.; 2016.

[ref17] Elam J. W.; Sechrist Z. A.; George S. M. ZnO/Al2O3 Nanolaminates Fabricated by Atomic Layer Deposition: Growth and Surface Roughness Measurements. Thin Solid Films 2002, 414, 43–55. 10.1016/S0040-6090(02)00427-3.

[ref18] Oehrlein G. S.; Metzler D.; Li C. Atomic Layer Etching at the Tipping Point: An Overview. ECS J. Solid State Sci. Technol. 2015, 4, N5041–N5053. 10.1149/2.0061506jss.

[ref19] George S. M. Mechanisms of Thermal Atomic Layer Etching. Acc. Chem. Res. 2020, 53, 1151–1160. 10.1021/acs.accounts.0c00084. 32476413

[ref20] Biyikli N.; Haider A. Atomic Layer Deposition: An Enabling Technology for the Growth of Functional Nanoscale Semiconductors. Semicond. Sci. Technol. 2017, 32, 093002 10.1088/1361-6641/aa7ade.

[ref21] Konh M.; Wang Y.; Chen H.; Bhatt S.; Xiao J. Q.; Teplyakov A. V. Selectivity in Atomically Precise Etching: Thermal Atomic Layer Etching of a CoFeB Alloy and Its Protection by MgO. Appl. Surf. Sci. 2022, 575, 151751 10.1016/j.apsusc.2021.151751.

[ref22] Konh M.; Janotti A.; Teplyakov A. Molecular Mechanism of Thermal Dry Etching of Iron in a Two-Step Atomic Layer Etching Process: Chlorination Followed by Exposure to Acetylacetone. J. Phys. Chem. C 2021, 125, 7142–7154. 10.1021/acs.jpcc.0c10556.

[ref23] George S. M.; Lee Y. Prospects for Thermal Atomic Layer Etching Using Sequential, Self-Limiting Fluorination and Ligand-Exchange Reactions. ACS Nano 2016, 10, 4889–4894. 10.1021/acsnano.6b02991. 27216115

[ref24] Sechrist Z. A.; Fabreguette F. H.; Heintz O.; Phung T. M.; Johnson D. C.; George S. M. Optimization and Structural Characterization of W/Al 2O 3 Nanolaminates Grown Using Atomic Layer Deposition Techniques. Chem. Mater. 2005, 17, 3475–3485. 10.1021/cm050470y.

[ref25] Conley J. F.; Alimardani N. Impact of Electrode Roughness on Metal-Insulator-Metal (MIM) Diodes and Step Tunneling in Nanolaminate Tunnel Barrier Metal-Insulator-Insulator-Metal (MIIM) Diodes. In Rectenna Solar Cells; Springer New York: New York, NY, 2013; Vol. 9781461437, pp 111–134.

[ref26] Hayrinen M.; Roussey M.; Gandhi V.; Stenberg P.; Saynatjoki A.; Karvonen L.; Kuittinen M.; Honkanen S. Low-Loss Titanium Dioxide Strip Waveguides Fabricated by Atomic Layer Deposition. J. Lightwave Technol. 2014, 32, 208–212. 10.1109/JLT.2013.2291960.

[ref27] Lau W. S.; Du L.; Yu D. Q.; Wang X.; Wong H.; Xu Y. The Application of a Selective Etch to Conclusively Show the Surface Smoothing Effect of an Amorphous Thin Film Deposited by Atomic Layer Deposition. ECS J. Solid State Sci. Technol. 2017, 6, N111–N116. 10.1149/2.0061708jss.

[ref28] Myers T. J.; Throckmorton J. A.; Borrelli R. A.; O’Sullivan M.; Hatwar T.; George S. M. Smoothing Surface Roughness Using Al2O3 Atomic Layer Deposition. Appl. Surf. Sci. 2021, 569, 150878 10.1016/j.apsusc.2021.150878.

[ref29] Lau W. S.; Zhang J.; Wan X.; Luo J. K.; Xu Y.; Wong H. Surface Smoothing Effect of an Amorphous Thin Film Deposited by Atomic Layer Deposition on a Surface with Nano-Sized Roughness. AIP Adv. 2014, 4, 027120 10.1063/1.4866988.

[ref30] Lau W. S.; Zhang J.; Wan X.; Wong H.; Luo J. K.; Xu Y. Surface Smoothing Effect of an Amorphous Thin Film Deposited by Chemical Vapor Deposition or Atomic Layer Deposition. ECS Trans. 2014, 60, 527–531. 10.1149/06001.0527ecst.

[ref31] Alasaarela T.; Korn D.; Alloatti L.; Säynätjoki A.; Tervonen A.; Palmer R.; Leuthold J.; Freude W.; Honkanen S. Reduced Propagation Loss in Silicon Strip and Slot Waveguides Coated by Atomic Layer Deposition. Opt. Express 2011, 19 (12), 11529 10.1364/OE.19.011529. 21716384

[ref32] Kanarik K. J.; Tan S.; Gottscho R. A. Atomic Layer Etching: Rethinking the Art of Etch. J. Phys. Chem. Lett. 2018, 9, 4814–4821. 10.1021/acs.jpclett.8b00997. 30095919

[ref33] Ohba T.; Yang W.; Tan S.; Kanarik K. J.; Nojiri K. Atomic Layer Etching of GaN and AlGaN Using Directional Plasma-Enhanced Approach. Jpn. J. Appl. Phys. 2017, 56, 06HB06 10.7567/JJAP.56.06HB06.

[ref34] Zywotko D. R.; Faguet J.; George S. M. Rapid Atomic Layer Etching of Al2O3 Using Sequential Exposures of Hydrogen Fluoride and Trimethylaluminum with No Purging. J. Vac. Sci. Technol. A 2018, 36, 061508 10.1116/1.5043488.

[ref35] Lee Y.; George S. M. Atomic Layer Etching of Al2O3using Sequential, Self-Limiting Thermal Reactions with Sn(Acac)2and Hydrogen Fluoride. ACS Nano 2015, 9, 2061–2070. 10.1021/nn507277f. 25604976

[ref36] Lee Y.; DuMont J. W.; George S. M. Atomic Layer Etching of HfO 2 Using Sequential, Self-Limiting Thermal Reactions with Sn(Acac) 2 and HF. ECS J. Solid State Sci. Technol. 2015, 4, N5013–N5022. 10.1149/2.0041506jss.

[ref37] Murdzek J. A.; George S. M. Effect of Crystallinity on Thermal Atomic Layer Etching of Hafnium Oxide, Zirconium Oxide, and Hafnium Zirconium Oxide. J. Vac. Sci. Technol. A 2020, 38, 022608 10.1116/1.5135317.

[ref38] Cano A. M.; Marquardt A. E.; Dumont J. W.; George S. M. Effect of HF Pressure on Thermal Al2O3 Atomic Layer Etch Rates and Al2O3 Fluorination. J. Phys. Chem. C 2019, 123, 10346–10355. 10.1021/acs.jpcc.9b00124.

[ref39] Chittock N. J.; Vos M. F. J.; Faraz T.; Kessels W. M. M. E.; Knoops H. C. M.; Mackus A. J. M. Isotropic Plasma Atomic Layer Etching of Al2O3 Using a Fluorine Containing Plasma and Al(CH3)3. Appl. Phys. Lett. 2020, 117, 162107 10.1063/5.0022531.

[ref40] Kim J.; Shim D.; Kim Y.; Chae H. Atomic Layer Etching of Al2O3 with NF 3 Plasma Fluorination and Trimethylaluminum Ligand Exchange. J. Vac. Sci. Technol. A 2022, 40, 032603 10.1116/6.0001616.

[ref41] Macco B.; Knoops H. C. M.; Verheijen M. A.; Beyer W.; Creatore M.; Kessels W. M. M. Atomic Layer Deposition of High-Mobility Hydrogen-Doped Zinc Oxide. Sol. Energy Mater. Sol. Cells 2017, 173, 111–119. 10.1016/j.solmat.2017.05.040.

[ref42] Jacobs T. D. B.; Junge T.; Pastewka L. Quantitative Characterization of Surface Topography Using Spectral Analysis. Surf. Topogr. 2017, 5, 013001 10.1088/2051-672X/aa51f8.

[ref43] Persson B. N. J.; Albohr O.; Tartaglino U.; Volokitin A. I.; Tosatti E. On the Nature of Surface Roughness with Application to Contact Mechanics, Sealing, Rubber Friction and Adhesion. J. Phys.: Condens. Matter 2005, 17, R1–R62. 10.1088/0953-8984/17/1/R01. 21690662

[ref44] Sethian J. A.; Adalsteinsson D. An Overview of Level Set Methods for Etching, Deposition, and Lithography Development. IEEE Trans. Semicond. Manuf. 1997, 10, 167–184. 10.1109/66.554505.

[ref45] Chacon A.; Vladimirsky A. A Parallel Two-Scale Method for Eikonal Equations. SIAM J. Sci. Comput. 2015, 37, A156–A180. 10.1137/12088197X.

[ref46] Shi S.; Qian S.; Hou X.; Mu J.; He J.; Chou X. Structural and Optical Properties of Amorphous Al2O3 Thin Film Deposited by Atomic Layer Deposition. Adv. Condens. Matter Phys. 2018, 2018, 7598978 10.1155/2018/7598978.

[ref47] Puurunen R. L. Random Deposition as a Growth Mode in Atomic Layer Deposition. Chem. Vap. Deposition 2004, 10, 159–170. 10.1002/cvde.200306283.

[ref48] Deal B. E.; Grove A. S. General Relationship for the Thermal Oxidation of Silicon. J. Appl. Phys. 1965, 36, 3770–3778. 10.1063/1.1713945.

[ref49] Kao D.-B.; McVittie J. P.; Nix W. D.; Saraswat K. C. Two-Dimensional Thermal Oxidation of Silicon. II. Modeling Stress Effects in Wet Oxides. IEEE Trans. Electron Devices 1988, 35, 25–37. 10.1109/16.2412.

[ref50] Kao D.-B.; McVittie J. P.; Nix W. D.; Saraswat K. C. Two-Dimensional Thermal Oxidation of Silicon—I. Experiments. IEEE Trans. Electron Devices 1987, 34, 1008–1017. 10.1109/T-ED.1987.23037.

[ref51] Honda M.; Katsunuma T.; Tabata M.; Tsuji A.; Oishi T.; Hisamatsu T.; Ogawa S.; Kihara Y. Benefits of Atomic-Level Processing by Quasi-ALE and ALD Technique. J. Phys. D: Appl. Phys. 2017, 50, 234002 10.1088/1361-6463/aa6f27.

[ref52] Lopes M. C. V.; dos Santos Fo S. G.; Hasenack C. M.; Baranauskas V. Si - SiO2 Electronic Interface Roughness as a Consequence of Si - SiO2 Topographic Interface Roughness. J. Electrochem. Soc. 1996, 143, 1021–1025. 10.1149/1.1836575.

[ref53] Vorburger T. V.; Marx E.; Lettieri T. R. Measurable With Light Scattering. Appl. Opt. 1993, 32, 3401–3408. 10.1364/AO.32.003401. 20829957

[ref54] Gertsch J. C.; Sortino E.; Bright V. M.; George S. M. Deposit and Etchback Approach for Ultrathin Al_2_O_3_ Films with Low Pinhole Density Using Atomic Layer Deposition and Atomic Layer Etching. J. Vac. Sci. Technol., A 2021, 39 (6), 062602 10.1116/6.0001340.

