# Supplementary material for: Surface Smoothing by Atomic Layer Deposition and Etching for the Fabrication of Nanodevices
Source: ACS Appl Nano Mater. 2022 Nov 28;5(12):18116–26. doi: 10.1021/acsanm.2c04025 (PMC9791650; doi:10.1021/acsanm.2c04025)
Supplement: Supplementary file 1 — an2c04025_si_001.pdf [file an2c04025_si_001.pdf]

Supporting information

Surface Smoothing by Atomic Layer Deposition and Etching for Fabrication of Nanodevices

Sven H. Gerritsen,[1] Nicholas J. Chittock[1], Vincent Vandalon[1], Marcel A. Verheijen[1,2], Harm C.M. Knoops[1,3], Wilhelmus M.M. Kessels[1] and Adriaan J.M. Mackus[1]**

AUTHOR ADDRESS

1. Department of Applied Physics, Eindhoven University of Technology, P.O. Box 513, 5600 MB Eindhoven, The Netherlands

2. Eurofins Materials Science, High Tech Campus 11, 5656AE Eindhoven, The Netherlands

3. Oxford Instruments Plasma Technology, North End, Bristol BS49 4AP, United Kingdom

*Email: A.J.M.Mackus@tue.nl,

N.J.Chittock@tue.nl

S.A Inherent roughness on smooth Si substrates

ALD of Al_2O_3 on a rough surface leads to smoothing, however when deposited on a very smooth surface, ALD of Al_2O_3 is observed to lead to an increase in roughness, as is shown in Figure S.1. In literature an increase in roughness due to ALD of Al_2O_3 has also been observed.[1,2] The observed roughness is not caused by crystallinity, as the deposited film is amorphous, instead, it has been hypothesized that the roughening effect is caused by random deposition.[3] The roughness that forms after ALD on a smooth surface, is inherent to the specific deposition process or process conditions (i.e., deposition temperature, purge times, dose times etc.) and is therefore referred to as the inherent roughness.

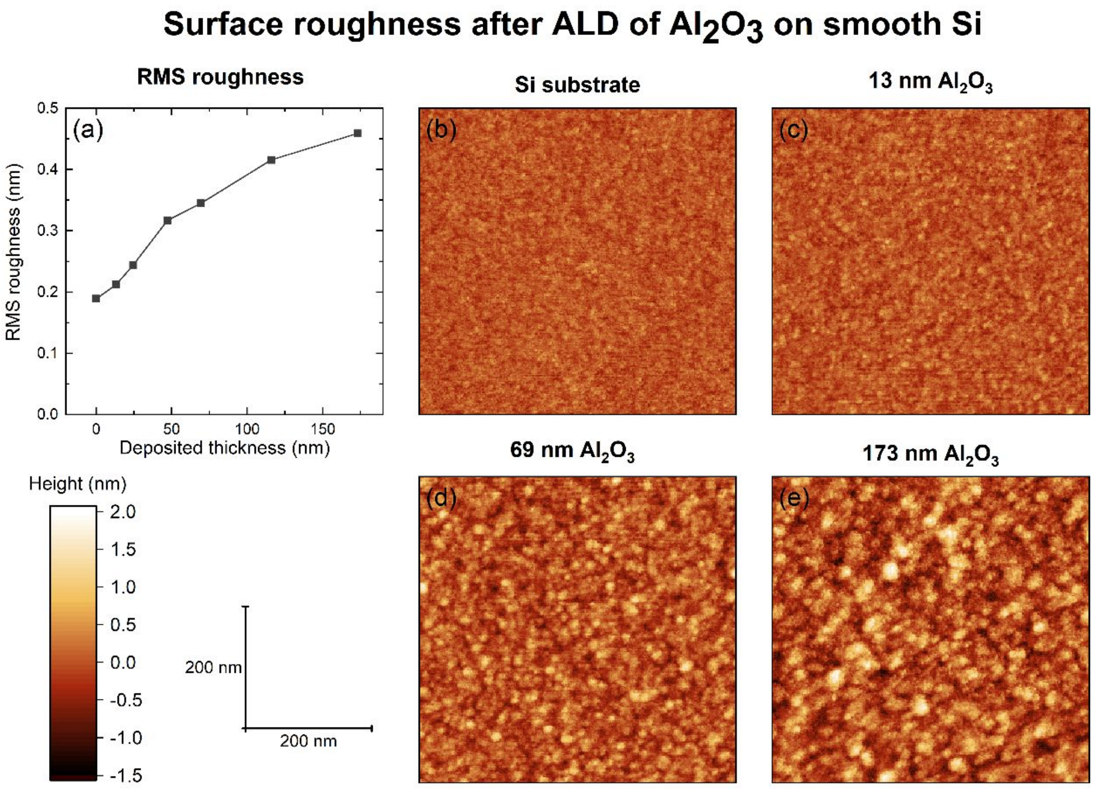

Figure S1. (a) RMS roughness after ALD of Al_2O_3 on Si as measured by AFM. (b)-(e) AFM height maps of different thicknesses of Al_2O_3 deposited on Si.

The heightmaps from the model and AFM in Figure S.2a and S.2b are similar. However, there is a difference in small-scale roughness. The cause of this difference is two-fold: firstly, the model does not include measurement noise, and secondly, the model does not include the inherent roughness of the Al_2O_3 film. These two effects can be corrected for by using the inherent roughness, as measured by AFM on a film of Al_2O_3 deposited on smooth Si. The measured inherent

roughness is added to the model results by adding the values of the height of the corresponding datapoints. This technique is applied to 173 nm ALD on 300 cycles of ZnO as is shown in Figure S.2. The sum of the model data and the inherent roughness is shown in Figure S.2c, which matches very closely to the experimental data, as shown in Figure S.2d. The same correction is shown in the form of the PSD in Figure S.3.

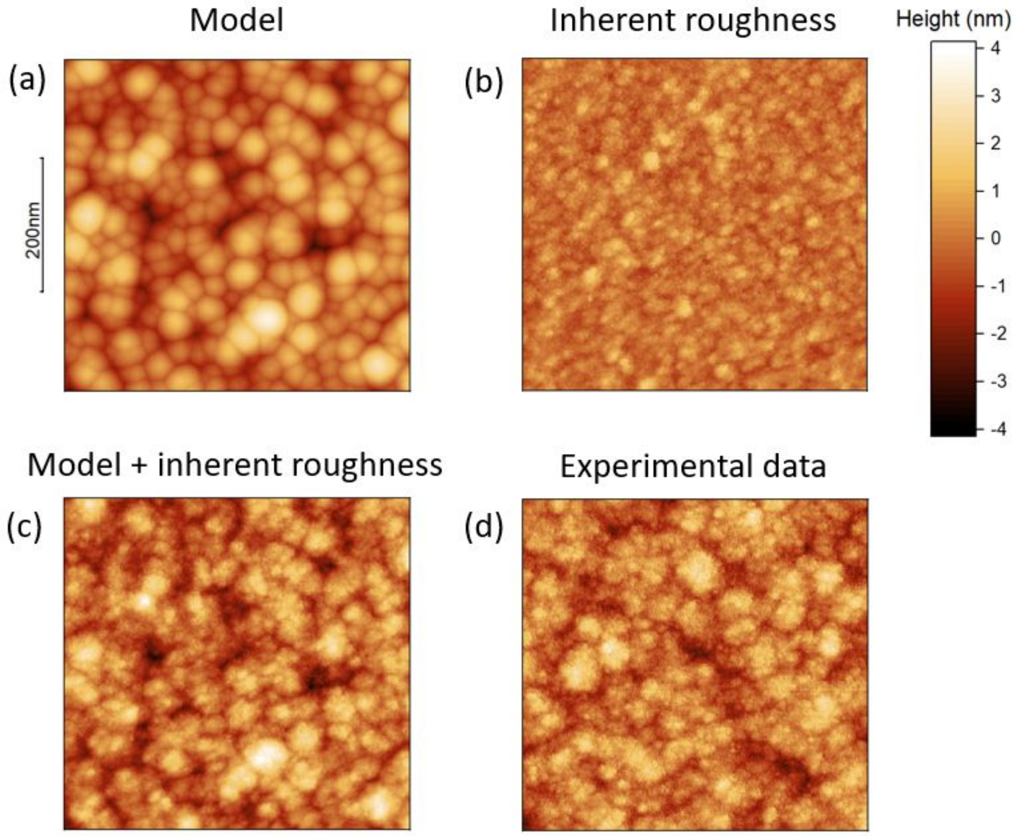

Figure S2. (a) Model result of 173 nm of Al_2O_3 deposited on 47 nm ZnO. (b) Roughness of 173 nm of Al_2O_3 as deposited using ALD on a smooth Si surface, as measured by AFM. The measured

roughness is the inherent roughness. (c) The sum of the (a) model results and (b) the inherent roughness. The roughness of 173 nm of Al_2O_3 deposited on 47 nm ZnO.

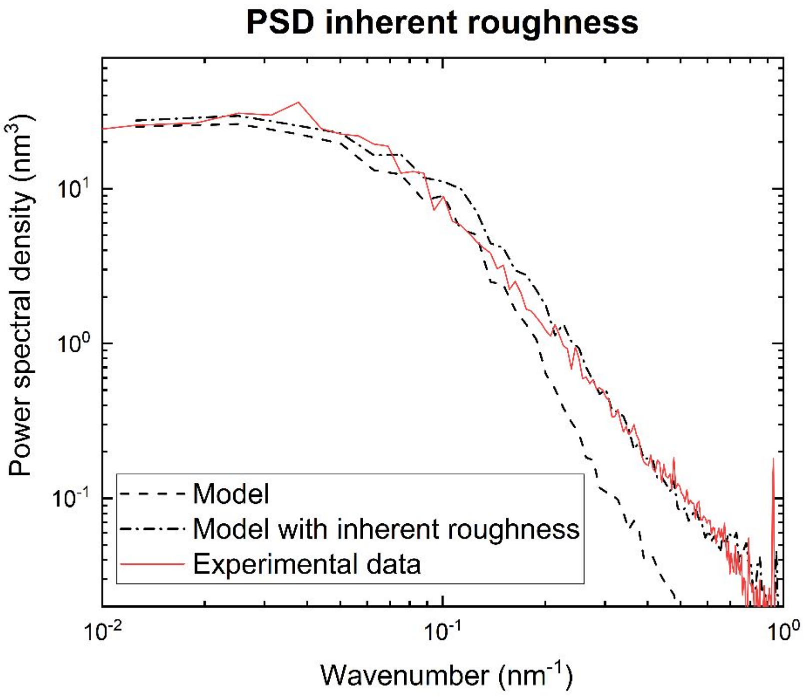

Figure S3. The model and experimental data deviate for high wavenumbers. When correcting for the inherent roughness of the ALD process, the model data better matches the experimental data.

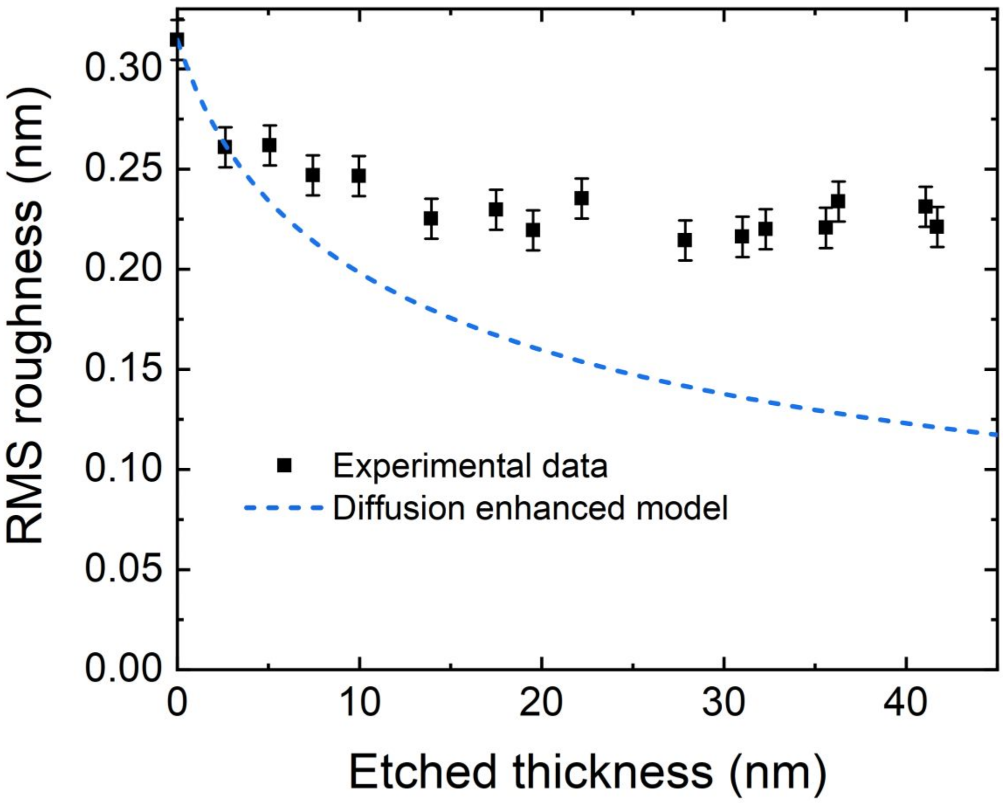

Figure S4. The RMS roughness after ALE of 61 nm of Al_2O_3 deposited on smooth Si. The roughness of the Al_2O_3 film is caused by the inherent roughness of the process. The RMS roughness decreases as a function of the etched thickness, and this decrease is fast at first but plateaus at a level of ~0.22 nm. After the first data point the results are not well described by the model using $\varepsilon=1.5 \cdot 10^{-9}$ m. The deviation could be explained by the roughness of the Si substrate underneath (RMS roughness 0.2 nm) or the inherent roughness of the ALE process. This was not investigated further.

S.B TEM roughness analysis

A TEM image can be converted into a 1D image (using Gwyddion software), resulting in plots of the mean pixel intensity for each line of pixels starting from the top of the image, as shown in Figure S.5. From this the rise/fall of pixel intensity at each interface can be calculated, which provides an approximation of the mean roughness, R_a. The mean roughness is calculated as

$$R_a = \frac{1}{L}\int_0^L |Z(x)|\mathrm{dx} \quad (1)$$

where L is the length over which roughness is evaluated, x a point along the length 0 → L and $Z(x)$ the height of the film at point x. R_a can be further approximated as

$$R_a = \frac{|Z_1| + |Z_2| \ldots + |Z_N|}{N} \quad (2)$$

with N being a unit step along a line and Z_N being the height at point N. For this analysis N is the width of the TEM image in number of pixels and Z the mean pixel intensity. Fitting the pixel intensity using a step function provides the width of the rise/fall region of each step, which approximates how abrupt(or smooth) an interface is. A rough film should have a wide rise/fall region whereas a smooth film should have a narrow region. Half of this rise/fall width is then roughly equivalent to the mean roughness. The values obtained from this method can be seen in

Table S.1. The analysis for layer 2 post ALD was not possible to obtain from the TEM analysis as it had been partly removed by ALE. The TEM method appears to overestimate the roughness of each interface, which is potentially due to the depth of field inherent in a TEM image, causing blurring of the interface.

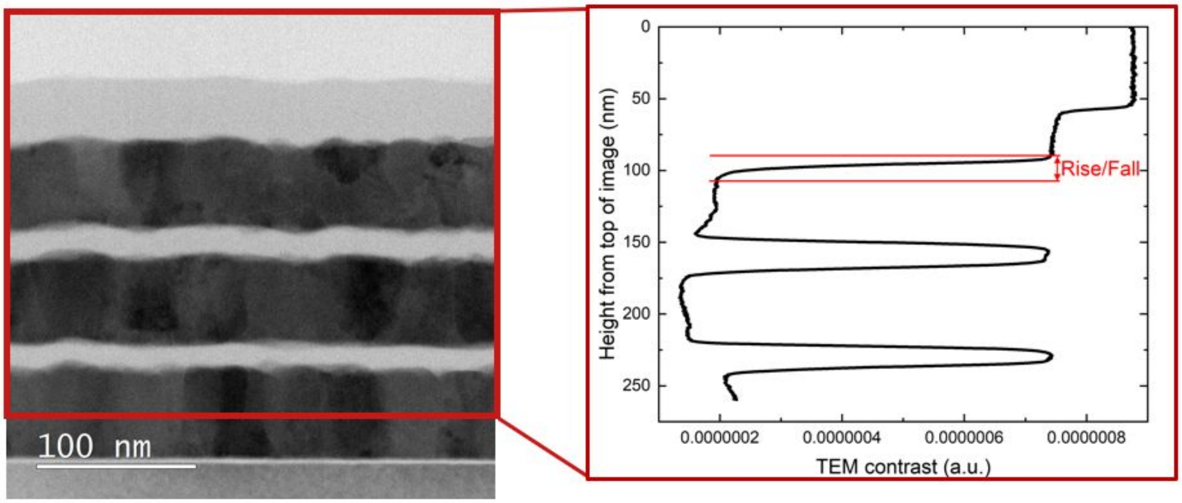

Figure S5. Example of TEM image and corresponding mean pixel intensity map. The red box highlights the region that has been converted into the pixel map shown on the right. The rise/fall parameter is also highlighted on the pixel intensity map.

S.C PSD noise correction

Surfaces as measured by AFM always have a certain amount of electronic noise. This noise can be corrected for by two methods. The first method is to measure the noise of the AFM measurement

itself, which is done by setting the measurement size to something very small (e.g., 1 nm). The AFM that is used cannot measure features this small, so only measurement noise is observed. The noise is assumed to be independent of the measurement area, meaning that for different measurement sizes, the PSD is identical. Subtracting the noise PSD from the measured PSD results in the noise-corrected PSD. The second method uses the fact that the high wavenumber tail of a PSD should have a constant gradient. To correct for this a constant level of PSD noise can be subtracted from the data such that the high wavenumber tail approximates a straight line. Due to the y axis being logarithmic the subtraction of this constant only becomes noticeable at the higher wavenumbers were noise plays a greater role.

In Figure S.6 the two different methods are illustrated. The first method is applicable for low roughness surfaces, where the PSD remains low over the entire wavenumber range, Figure S.6a. While for high roughness surfaces the second method is most appropriate, where the noise from the AFM measurement is negligible, as can be seen from the good agreement at low wavenumber in Figure S.6b, and noise only has an impact at high wavenumbers. The first method was thus used for the ALE cases, as the roughness was generally lower. The second method was used for the ALD cases, in which instance a value of $1 \cdot 10^{-28}$ m^{-3} gives the best results.

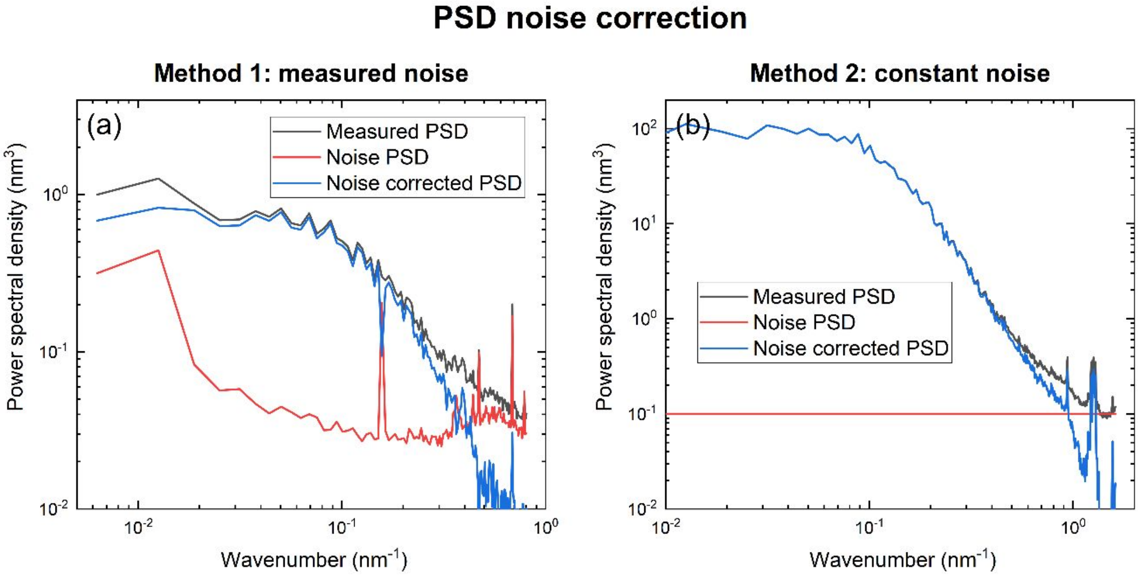

Figure S6. (a) Illustation of method 1. The measured PSD is that of a surface after 20 nm of ALE on 60 nm of Al_2O_3 as deposited on smooth Si. After noise correction, the PSD is significantly lower for high wavenumbers, as electronic noise mostly effects the small-scale features. (b) Method 2 using a constant value of the PSD. The measured PSD is that of a 47 nm of ZnO as deposited by ALD. After noise correction the PSD more closely approximates a straight line for have wavenumber values, which is to be expected.

S.D Model

The basis of the deposition and etch model lies in the work by Sethian *et al.*, [4–6] in which a propagating front is modelled using the eikonal equation, which models wave propagation, and is often used in optics. The eikonal equation is given by

$$|\nabla T|F = 1 \qquad (3)$$

with F the propagation rate. The front is defined as the level set for which $T(x,y,z)=\tau$, with τ the amount of deposited or etched material (positive τ for deposition and negative τ for etching). In this work only surfaces without overhang are considered, meaning that squaring equation 3 and multiplying by $(\partial z/\partial T)^2$ gives the following equation

$$\left(\frac{\partial z}{\partial T}\right)^2 = F^2\left(1 + \left(\frac{\partial T}{\partial x}\right)^2 + \left(\frac{\partial T}{\partial y}\right)^2\right), \qquad (4)$$

For the front T=τ, and since the surface has no overhangs $z=h(x,y)$, the equation can be reduced to

$$\frac{\partial h}{\partial \tau} = F\sqrt{1 + |\nabla h|^2}\,. \qquad (5)$$

For an ideally conformal ALD process, or an ideally isotropic etch process, the propagation rate F is equal to 1, which gives the equation from the work of Alasaarela *et al.*.[7] In this case the front propagation is monotonic/isotropic/uniform, i.e. equal everywhere on the surface.

For the ALE process studied in this work, the propagation is not uniform, meaning that *F* is not constant, but is dependent on the mean curvature *K(x,y)* by *F=1-εK*. The mean curvature is the mean of the two principle curvatures. Using Euler's theorem (of differential geometry), it can be proven that the mean curvature is equal to the mean of any two orthogonal curvatures (*x* and *y* planes in this case).

$$K_x = \frac{\partial^2 h}{\partial x^2}\left(1 + \left(\frac{\partial h}{\partial x}\right)^2\right)^{-\frac{3}{2}} \qquad (6)$$

$$K_y = \frac{\partial^2 h}{\partial y^2}\left(1 + \left(\frac{\partial h}{\partial y}\right)^2\right)^{-\frac{3}{2}}. \qquad (7)$$

S.E Smoothing rate for ZnO/Al_2O_3 stack

Smoothing rate values were also determined for the dataset presented in section 4.3, as listed in Table S.2. Interestingly, this shows a different trend in that the ALE smoothing is not necessarily the fastest as was seen for the thicker deposition/etch. The rates of smoothing for layer 2 (35 nm ALD), 2 (20 nm ALE), 4 (15 nm ALD) and 6 (35 nm ALD) can be seen in table S.1, showing that the fastest smoothing was observed for layer 4. A possible explanation for layer 4 having the highest rate is that the deposition was on the roughest ZnO layer, thus had the most roughness to smooth. Comparing both 35 nm ALD layers, we see that the rate for layer 6 is nearly double that

of layer 2 (ALD), which again could be explained by the higher starting roughness for layer 6. This confirms that when depositing very thin layers, the roughness of the starting surface plays an important role in the rate of smoothing that is achieved.

Table S1. Process used for each layer deposited/etched in Figure 7 with the film thickness from ellipsometry, RMS roughness and smoothing rate from AFM, and the roughness determined from TEM.

Layer	Process	Layer Thickness (nm)	RMS Roughness, R_{RMS} (nm)	Roughness reduction from ZnO layer (nm)	Rate of smoothing ($x10^{-3}$ nm/nm)	TEM roughness (nm)	TEM standard deviation (± nm)
1	55 nm ALD ZnO	55.35	1.51	-	-	6.87	0.49
2 (Post ALD)	35 nm ALD Al_2O_3	34.14	1.29	0.22	6.3	-	-
2 (Post ALE)	20 nm ALE Al_2O_3	14.15	0.98	0.53	15.5	5.23	0.58
3	55 nm ALD ZnO	54.97	2.13	-	-	7.69	0.60

4	15 nm ALD Al2O3	15.81	1.84	0.29	18.3	6.76	0.52
5	55 nm ALD ZnO	52.23	1.81	-	-	7.70	0.69
6	35 nm ALD Al_2O_3	36.13	1.38	0.42	11.6	6.43	0.69

S.F AFM maps post ALE

Similar to ALD the uniform front propagation + curvature dependent (UFP+CD) can be used to predict how a surface will smooth during ALE. Using a measured starting surface as the input for the model it can be seen that there is good agreement between the modelled and experimental data.

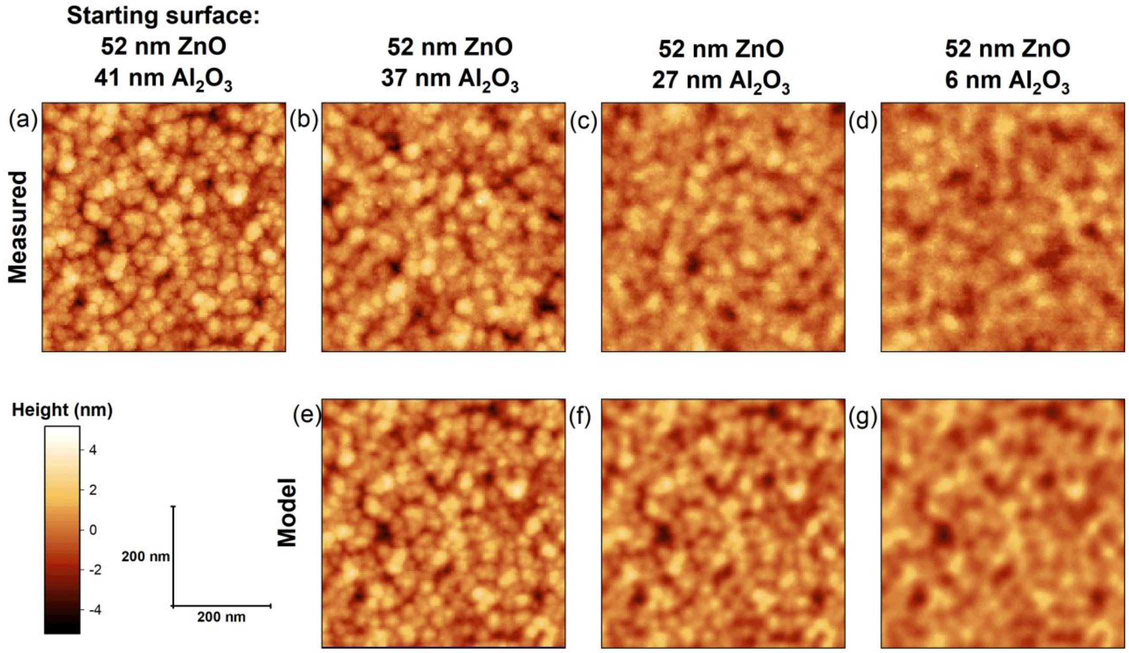

Figure S7. (a) AFM heightmap of the sample prepared by 400 cycles Al_2O_3 on 300 cycles ZnO, which is used as the starting surface for the experiments and model. (b)-(d) AFM heightmaps measured after ALE using SF_6 plasma and TMA. (e)-(g) Heightmaps from the UFP+CD model using ε=1.5·10^{-9} m, for films of the same thickness as in (b-d)

S.G Optical properties of Al_2O_3

Optical constant n is shown for a 35 nm Al_2O_3 film grown at 300 ˚C fitted using a Cauchy model. The extinction co-efficient k was zero over the entire wavelength range investigated here

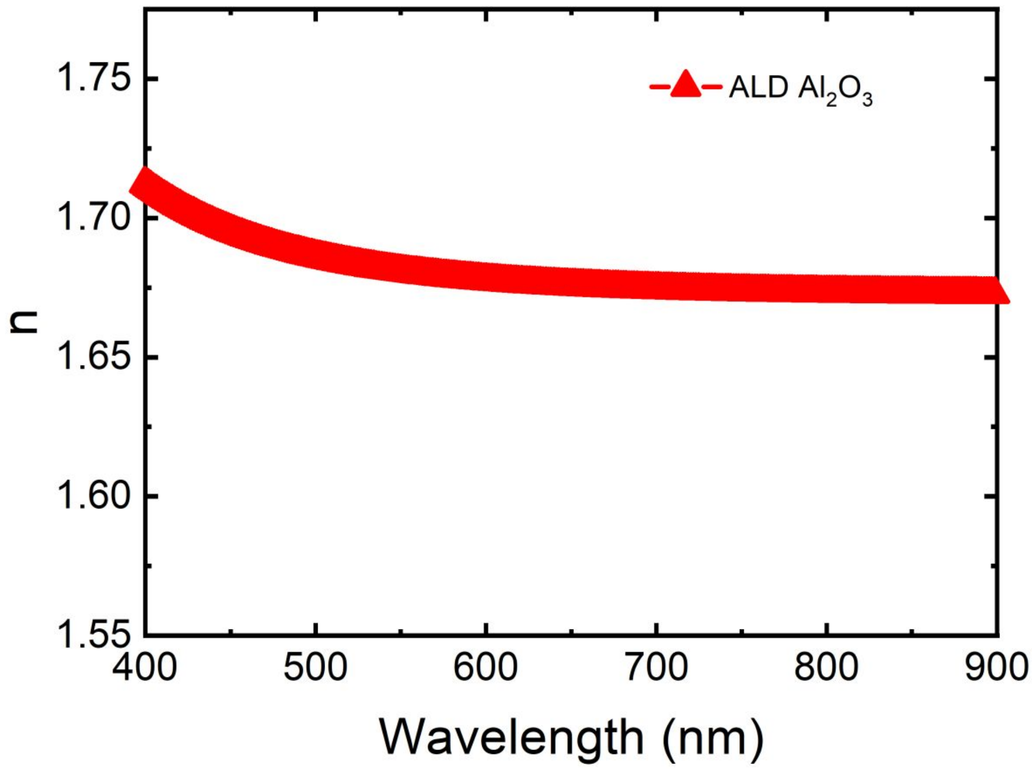

Figure S8: Refractive index for plasma ALD grown Al_2O_3.

REFERENCES

1. Shi, S. *et al.* Structural and optical properties of amorphous Al2O3 thin film deposited by atomic layer deposition. *Advances in Condensed Matter Physics* **2018**, (2018).

2. Elam, J. W., Sechrist, Z. A. & George, S. M. ZnO/Al2O3 nanolaminates fabricated by atomic layer deposition: Growth and surface roughness measurements. *Thin Solid Films* **414**, 43–55 (2002).

3. Jacobs, T. D. B., Junge, T. & Pastewka, L. Quantitative characterization of surface topography using spectral analysis. *Surface Topography: Metrology and Properties* **5**, (2017).

4. Sethian, J. A. Communications in Mathematical Curvature and the Evolution of Fronts. *Commun. Math. Phys* **101**, 487–499 (1985).

5. Sethian, J. A. & Adalsteinsson, D. An overview of level set methods for etching, deposition, and lithography development. *IEEE Transactions on Semiconductor Manufacturing* **10**, 167–184 (1997).

6. Adalsteinsson, D. & Sethian, J. A. A Level Set Approach to a Unified Model for Etching, Deposition, and Lithography I: Algorithms and Two-Dimensional Simulations. *Journal of Computational Physics* vol. 120 128–144 Preprint at https://doi.org/10.1006/jcph.1995.1153 (1995).

7. Alasaarela, T. *et al.* Integrated optics materials; (220.4241) Nanostructure fabrication; (230.7370) Waveguides; (240.5770) Roughness; (310.2785) Guided wave applications. **19**, 11529–11538 (2011).
